# Beyond fur color: differences in socio-emotional behavior and the oxytocin system between male BL6 and CD1 mice in adolescence and adulthood

**DOI:** 10.3389/fnins.2024.1493619

**Published:** 2024-12-09

**Authors:** Katharina Gryksa, Theresa Schäfer, Franziska Gareis, Elena Fuchs, Melanie Royer, Anna K. Schmidtner, Anna Bludau, Inga D. Neumann

**Affiliations:** Department of Behavioral and Molecular Neurobiology, University of Regensburg, Regensburg, Germany

**Keywords:** neuropeptide, mouse strain, anxiety-related behavior, fear conditioning, social avoidance, development, stress susceptibility, pain perception

## Abstract

**Introduction:**

The development of stress-related psychopathologies, often associated with socio-emotional dysfunctions, is crucially determined by genetic and environmental factors, which shape the individual vulnerability or resilience to stress. Especially early adolescence is considered a vulnerable time for the development of psychopathologies. Various mouse strains are known to age-dependently differ in social, emotional, and endocrine stress responses based on genetic and epigenetic differences. This highlights the importance of the qualified selection of an adequate strain and age for any biomedical research. Neuropeptides like oxytocin (OXT) can contribute to individual and strain-dependent differences in emotional and social behaviors.

**Methods:**

In this study, we compared anxiety- and fear-related, as well as social behavior and pain perception between male adolescent and adult mice of two commonly used strains, C57BL/6N (BL6) and CD1.

**Results:**

We revealed BL6 mice as being more anxious, less social, and more susceptible toward non-social and social trauma, both in adolescence and adulthood. Furthermore, during development from adolescence toward adulthood, BL6 mice lack the reduction in fear- and anxiety-related behavior seen in adult CD1 mice and show even higher social fear-responses and perception of noxious stimuli during adulthood. Analysis of the OXT system, by means of receptor autoradiography and immunohistochemistry, showed strain- and age-specific differences in OXT receptor (OXTR) binding in relevant brain regions, but no differences in the number of hypothalamic OXT neurons. However, intracerebroventricular infusion of OXT did neither reduce the high level of anxiety-related nor of social fear-related behavior in adult BL6 mice.

**Discussion:**

In summary, we show that male BL6 mice present an anxious and stress vulnerable phenotype in adolescence, which further exacerbates in adulthood, whereas CD1 mice show a more resilient socio-emotional state both in adolescence as well as during adulthood. These consistent behavioral differences between the two strains might only be partly mediated by differences in the OXT system but highlight the influence of early-life environment on socio-emotional behavior.

## Introduction

In humans and rodents alike, the genetically determined biological predisposition to either stress vulnerability or stress resilience, as well as environmental factors affect the development of stress-related psychopathologies, which are often associated with socio-emotional dysfunctions (Bludau et al., 2019; Broekman, 2011; Niitsu et al., 2019). Animal models reflecting trait differences in anxiety, stress coping, or social behaviors frequently describe varying susceptibility between individuals, rodent strains and developmental stages, and are valuable tools to reveal underlying genetic, physiological, cellular, or molecular mechanisms (Gryksa et al., 2023; Hodes et al., 2014; Langgartner et al., 2017; Swaminathan et al., 2023; Wegener et al., 2012).

Innate differences in stress vulnerability and their multiple consequences have frequently been studied in two disparate mouse strains – inbred C57BL/6N (BL6) and outbred CD1. Compared to CD1 mice, BL6 mice display higher anxiety-related behavior, e.g., in the novel open space test (Michalikova et al., 2010) and the rat exposure test (Yang et al., 2004). In the latter paradigm, BL6 mice displayed higher freezing and risk assessment behavior in conjunction with lower contact to the rat. Likewise, BL6 mice showed heightened passive stress coping and defensive behavior during exposure to chronic psychosocial stress (chronic subordinate colony housing; CSC) compared CD1 mice (Gryksa-Zotz, 2022).

Further substantial differences between the two strains were identified in terms of immunological adaptations: Compared to CD1 mice, (i) male BL6 mice showed higher levels of pro-inflammatory cytokines and colonic inflammation in response to CSC exposure (Füchsl et al., 2014; Reber et al., 2016), (ii) female BL6 showed an increased susceptibility to bisphenol A-induced pyometra (Kendziorski et al., 2012), and (iii) male and female BL6 mice presented lower immune tolerance in response to an immune challenge during placental and fetal limb development (Prater et al., 2006).

Interestingly, within the inbred C57BL/6 strain, the genetically distinct substrains C57BL/6N (here BL6) and C57BL/6J (BL6J) (Simon et al., 2013) also vary in emotional behavior, physical fitness, and stress vulnerability (Kang et al., 2019; Simon et al., 2013; Sturm et al., 2015). In more detail, BL6 mice displayed higher trait anxiety on the elevated plus-maze (EPM), reduced social interaction (Matsuo et al., 2010; Simon et al., 2013), a higher susceptibility toward hyperthermia-induced seizures (Kang et al., 2019), and chronic glucocorticoid-induced depressive-like behavior (Sturm et al., 2015), compared to BL6J. Due to these behavioral differences between BL6J and BL6, we chose the BL6 mouse strain for the comparison with CD1 mice in our study.

Although laboratory mice, e.g., BL6 and CD1, are highly social, subtle differences in social behavior were described. For example, male BL6 mice generally present lower aggressive and territorial behavior, which is accompanied by lower basal testosterone, but higher corticosterone levels, compared to other strains including CD1 (Toth and Neumann, 2013; van Loo et al., 2003a; van Loo et al., 2003b; Weber et al., 2022). In line, male BL6 mice also displayed lower levels of social motivation following repeated social exposure compared to CD1 males (Toth et al., 2013). Strain differences in social behavior also exist in females. After social isolation, both adolescent and adult female BL6J mice, preferred food over social interactions, whereas CD1 favored social interactions (Ramsey et al., 2021). In support, lactating BL6 mice showed lower maternal aggressive behavior toward male intruders compared to CD1 dams (Gryksa et al., 2020).

In conclusion, BL6 mice are more stress vulnerable, more anxious, less socially motivated and less aggressive in comparison to CD1 mice.

Although seemingly at the extreme ends of a continuum between high and low stress susceptibility and vulnerability to socio-emotional stressors, and trauma, both inbred BL6 and outbred CD1 have proven to be suitable strains for basic research (Hsieh et al., 2017; Tuttle et al., 2018; van Loo et al., 2003a; van Loo et al., 2003b; Weber et al., 2022).

Striking individual differences in stress susceptibility have already been observed in adolescence - a highly vulnerable developmental period (Boyce, 2016; McCormick et al., 2017; Swaminathan et al., 2023). Acute or chronic stress exposure in adolescence was found to result in long-lasting neuronal, neuroendocrine, and behavioral maladaptations both in humans and rodents (de Araújo Costa Folha et al., 2017; McCormick et al., 2017; Sisk and Gee, 2022). Thus, in humans, substantial stress exposure in adolescence increases the risk for psychopathologies, such as anxiety disorders and social phobia, in stress vulnerable individuals (Blakemore et al., 2010; Kessler et al., 2007; Polanczyk et al., 2015). Therefore, the analysis of behavioral and/or physiological differences and their molecular underpinnings between adolescent BL6 and CD1 mice seems crucial to increase our understanding of individual stress susceptibility in adolescence.

One factor that might contribute to differences in fear-related behavior following fear conditioning is the perception of pain. Previous studies showed strain-, sex- and bodyweight-dependent differences in pain-related behavior in mice (Domínguez-Oliva et al., 2022; Smith, 2019). Here, we used the Hargreave’s Plantar Test (HPT) (Cheah et al., 2017; Deuis et al., 2017), and analyzed the animal’s sensitivity to foot shocks of various intensities (Foot shock sensitivity comparison, FSSC) (Nielsen and Crnic, 2002) to compare the perception of pain between CD1 and BL6 mice.

Additionally, neuropeptides, such as arginine vasopressin, corticotropin-releasing factor (CRF), neuropeptide S (NPS), and oxytocin (OXT) are particularly relevant for shaping emotional and social behaviors (Grinevich and Neumann, 2021; Gryksa et al., 2023; Hostetler and Ryabinin, 2013; Jurek and Neumann, 2018; Menon and Neumann, 2023; Shemesh et al., 2016; Slattery et al., 2015). For example, OXT exerts profound anxiolytic (Neumann and Slattery, 2016), fear-reducing (Knobloch et al., 2012; Menon et al., 2018; Toth et al., 2012a; Zoicas et al., 2014), anti-stress (Neumann et al., 1999; Neumann et al., 2000) and pro-social (Froemke and Young, 2021; Lukas et al., 2011) effects. Interestingly, induction of social fear affected the OXT system by increasing OXT receptor (OXTR) binding in various brain regions, like the lateral septum (LS) and central amygdala (CeA) (Zoicas et al., 2014). Of note, affecting OXT signaling during early life, either via OXT treatment or by decreasing neuronal ablation, has not only acute but also long-term effects on adult physiology and socio-emotional behavior (Carter et al., 2009; Nunes et al., 2021; Vaidyanathan and Hammock, 2017).

In the present study, we performed a complex analysis of socio-emotional behavior of male BL6 and CD1 mice both in adolescence and adulthood. Given the described behavioral phenotype of BL6 mice and the anxiolytic, pro-social and stress-buffering effects of OXT (Froemke and Young, 2021; Jurek and Neumann, 2018; Menon and Neumann, 2023), we tested the hypothesis, whether differences in the brain OXT system may contribute to the observed distinct behaviors between the strains. To this end, we compared we compared OXTR binding in relevant brain regions, i.e., the LS, the amygdala, the ventromedial hypothalamus (VMH) and periaqueductal gray (PAG), which play an essential role in social (Menon et al., 2022), and anxiety- and fear-related (Zhao et al., 2009) behaviors, as well as the amount OXT-positive cells within the PVN and SON between the two mouse strains. Moreover, we centrally applied OXT to BL6 mice and assessed its behavioral impact.

## Materials and methods

### Animals and husbandry

For all experiments (see panel A in respective Figures for experimental timeline), male BL6 and CD1 mice were purchased from Charles River (Sulzfeld, Germany) either at the age of 22–24 days (adolescent) or 8 weeks (adults). To avoid putative stressful influences between individuals of the two strains, all cages were placed in the same room, but strains were spatially separated to avoid visual and auditory contact. Adult mice remained group-housed in groups of 4 for 1 week to habituate to the new environment. To avoid the formation of a social hierarchy within the cage, mice were single-housed for the consecutive week, which was found to be least stressful (Kappel et al., 2017; Singewald et al., 2009; Smolensky et al., 2024). Adolescent mice remained group-housed in groups of 4 for 3 days and were single-housed for 2 days prior to behavioral assessment. BL6 and CD1 mice of the same age were tested in mixed order on the same day. For all behavioral tests involving social interactions, mice were placed in observation cages (30 cm × 25 cm × 35 cm) 2–7 days prior to testing, and weight and strain-matched mice were used as social stimuli. All mice were kept under standard laboratory conditions (22 ± 2°C, 50% humidity, 12-h light/dark cycle) with water and food *ad libitum*. All behavioral experiments were carried out between 08:00 am and 12:00 pm. Experiments were performed in accordance with the Guide for the Care and Use of Laboratory Animals by the National Institutes of Health, Bethesda, USA, approved by the government of Unterfranken and performed according to international guidelines on the ethical use of animals and ARRIVE guidelines (Kilkenny et al., 2010).

### Elevated plus-maze (EPM)

The EPM was performed to assess anxiety-related behavior and locomotor activity (Slattery et al., 2012). Briefly, the EPM consists of a plus-shaped elevated maze (70 cm height) with two closed (opaque walls; 50 cm × 10 cm, 10lux) and two open (50 cm × 10 cm, 50lux) arms connected by a neutral zone. Each mouse was placed into the neutral zone of the EPM facing a closed arm and allowed to explore the EPM for 5 min. The percentage of time spent on the open arms and the percentage of open arm entries (indicators of anxiety-related behavior), as well as the total number of closed arm entries (indicator of locomotion) were measured by a trained observer blind to treatment using JWatcher (V 1.0, Macquarie University and UCLA). Between testing, the apparatus was cleaned thoroughly with water containing soap and dried.

### Open field test (OFT)

The OFT was performed to assess locomotor activity and anxiety-related behavior as described earlier (Seibenhener and Wooten, 2015). In brief, within the OFT arena (40 cm × 40 cm × 38.5 cm; 300lux) an outer and center zone (20 cm × 20 cm) were defined. The experimental mouse was placed in the center of the arena and allowed to freely explore for 5 min. Behavior was videotaped and analyzed automatically using EthoVision XT7. The total distance moved was used as an indicator of locomotion, whereas the time spent in the center zone was used as indicator of anxiety-related behavior. Between testing, the apparatus was cleaned thoroughly with water containing soap and dried.

### Social preference/avoidance test (SPAT)

To analyze strain differences in naturally occurring social preference behavior the SPAT was performed (Lukas et al., 2011) in the homecage of the experimental mouse to reduce stressful environmental changes. In brief, mice were exposed to a non-social stimulus (empty wire-mesh cage) for 2.5 min, which was replaced by a social stimulus (wire-mesh cage containing an unknown age, weight, and strain-matched conspecific for another 2.5 min). The order of stimulus presentation remained the same for all exposed animals. The time spent investigating the non-social and social stimuli was analyzed by a trained observer blind to treatment (OXT or VEH) using JWatcher. Social preference was defined when mice investigated the social stimulus significantly longer than the object stimulus (Lukas et al., 2011).

### Cued fear conditioning (CFC)

CFC was performed in a computerized fear conditioning system (TSE System GmbH, Germany) (Toth et al., 2012a) in order to assess strain differences in fear learning and fear expression. The conditioning chamber consisted of a transparent Perspex box (45 cm × 22 cm × 40 cm) enclosed in a chamber to reduce external noise and visual stimulation. The floor was made of a removable stainless-steel grid, which was connected to a shock delivery unit to allow application of foot shocks. To perform CFC in distinct environments, different surroundings, floors, odors and light conditions were used for acquisition, extinction and recall. Freezing behavior was measured automatically using infrared beams. Between testing, the apparatus was cleaned thoroughly with water containing soap and dried.

On day 1, mice were first subjected to 5 min of habituation to the conditioning box followed by four exposures to a 30-s tone (conditioned stimulus—CS; 80 dB, 8 kHz) co-terminated with a 2-s mild foot shock (unconditioned stimulus—US; 0.7 mA, pulsed current). The CS-US coupling was applied at a 2-min interstimulus interval. On day 2 during fear extinction, 20 repetitions of the 30-s CS with a 5-s interstimulus interval were performed. For analysis, the freezing duration during two CS-presentations was summarized to one data point resulting in 10 data points. On day 3 during retention, mice were exposed to 2 repetitions of the 30-s CS with a 2-min interstimulus interval. Here, the freezing duration of both CS-presentations was summarized.

### Social fear conditioning (SFC)

SFC was performed to compare trauma-induced social fear between mouse strains using the TSE system (Toth et al., 2012b, 2013). On day 1 during fear acquisition, mice were individually placed in the conditioning chamber, and after a 30-s habituation period, they were exposed to an empty wire-mesh cage (non-social stimulus, 7 cm × 7 cm × 6 cm) for 3 min, which they freely investigated. Subsequently, the empty cage was replaced with an identical cage containing an unknown age, weight, and strain-matched conspecific (conditioned stimulus: CS). Conditioned mice (SFC^+^) received a mild foot shock (unconditioned stimulus: US; 0.7 mA, approx. 1 s) each time they investigated the conspecific. Unconditioned mice (SFC^−^) were allowed to freely interact with the conspecific for 3 min. SFC^+^ mice avoided approaching or interacting with the conspecific after 1–3 CS-US pairings, at which point they were considered to have acquired social fear and returned to their home cage. Between animals, the apparatus was cleaned thoroughly with water containing soap and dried. On day 2 during social fear extinction training, each single-housed mouse was exposed three times to an empty wire-mesh cage and then to six different age, weight, and strain-matched unknown conspecifics for 3 min each with a 3-min inter-exposure interval. The time spent investigating the social stimulus was measured as an indicator of social fear. On day 3 during recall, mice were again consecutively exposed to six different age, weight, and strain-matched social stimuli in their home cage for 3 min with a 3-min inter-exposure interval to assess short-term social fear extinction behavior. The investigation duration of each non-social and social stimuli by SFC^+^ and SFC^−^ mice was manually scored by an observer blind to treatment using JWatcher and is shown as percentage of time spent in direct contact.

### Foot shock sensitivity comparison (FSSC)

To compare foot shock perception, the response to different foot shock intensities was tested. On day 1, mice were weighted and placed in the transparent chamber (white light, 300lux, 23 cm × 23 cm × 36 cm) of a computerized fear conditioning system (TSE System GmbH, Germany). After 2 min of habituation, the following foot shocks were presented in identical order and a semi-randomized inter-shock interval of 30–60s: 0.05 mA, 0.1 mA, 0.15 mA, 0.2 mA, 0.3 mA, 0.5 mA, 0.7 mA, and 1.0 mA (pulsed current, 1 s duration). Between testing, the chamber was cleaned thoroughly with water containing lemon-scented soap and dried. The animal’s responses were scored using the following response scores: 0: no response (normal activity with no visible reaction to the shock); (1) flinch (jerky movement or abrupt body posture shift, remaining in the same location, three paws on the grid); (2) hop (small forward or backward horizontal—less than ½ of the chamber—or vertical—less than bodyheight—with at least two paws on the grid); (3) run (forward horizontal movement—greater than ½ of the chamber) with at least two paws on the grid; (4) horizontal jump (horizontal movement—greater than ½ of the chamber) with all four paws off the grid in a springing motion (bodyheight or higher); (5) vertical jump (vertical movement—greater than bodyheight—with all four paws off the grid) (Nielsen and Crnic, 2002). Further, the vocalization of the animal during each foot shock was scored. On day two, animals were exposed to the same procedure within a black chamber (white light, 300lux) and between testing, the chamber was cleaned thoroughly with water containing neutral-scented soap and dried.

### Hargreave’s plantar test (HPT)

To further compare pain perception, the HPT was performed as described previously (Hargreaves et al., 1988; Menon et al., 2018). Mice were habituated in the transparent plantar test chamber (8 × 6 × 6 cm; glass floor, Ugo Basile, Italy; 37,370) for 10 min on three consecutive days. On the test day, mice were weighted and placed in the test chamber and habituated for 10 min. Subsequently, a focused thermal stimulus (245 mW/cm^2^) was placed to the plantar surface of the hind paw and the paw withdrawal latency was automatically measured. Each hind paw was tested three times. Between testing, the chamber was cleaned thoroughly with water containing soap and dried.

### Stereotactic implantation of a guide cannula and intracerebral substance infusion

The implantation of a guide cannula (21G, 8 mm; Injecta GmbH, Germany; from Bregma +0.2 mm, lateral +1.0 mm, depth − 1.4 mm) (Paxinos and Franklin, 2019) for subsequent intracerebroventricular (icv) infusions was performed as previously described (Zoicas et al., 2014). In brief, mice were deeply anesthetized (2% Isoflurane, Forene; Abbott GmbH, Wiesbaden, Germany), and the cannula was fixed to the skull using two jeweler’s screws and dental cement (Kallocryl; Speiko-Dr. Speier GmbH, Münster, Germany). A stainless steel stylet (26G, 8 mm) was inserted into the guide cannula to avoid infections. To avoid postsurgical infections and pain, mice received a subcutaneous injection of the antibiotic Baytril (10 mg/kg Enrofloxacin, Bayer GmbH, Germany) and the analgesic Buprenovet (0.1 mg/kg Buprenorphine; Bayer), as well as the local anesthetic Lidocaine (Lidocainhydrochlorid 2%; Bela-pharm). Mice were allowed to recover for at least 7 days and were daily handled, before they were exposed to the first behavioral test.

20 min prior to exposure to the EPM and SPAT, or 10 min prior to SFC extinction training, mice received an icv infusion of either vehicle (Veh, 2 μL sterile Ringer solution), or synthetic OXT (low dose: 0.1 μg/2 μL; high dose: 0.5 μg/2 μL; Sigma Aldrich Chemie GmbH, Schnelldorf, Germany) using a stainless-steel infusion cannula (26G, 10 mm) inserted into the guide cannula and connected via a polyethylene tubing to a Hamilton syringe (Hamilton Company, Bonaduz, Switzerland). Cannula placement was histologically verified *post mortem* by infusing ink icv to visualize the ventricular system.

### OXTR autoradiography

To compare OXTR binding in specific brain areas of BL6 and CD1 mice, brains were snap frozen and stored at −20°C until coronal cryosectioning (16 μm) targeting the LS, CeA and basolateral (BLA) amygdala, VMH, and PAG. These regions were selected based on their role in conditioned fear and social behavior (Jurek and Neumann, 2018; LeDoux and Muller, 1997). OXTR autoradiography was performed as previously described (Lukas et al., 2010; Zoicas et al., 2014) using a linear OXTR antagonist [125I]-d(CH2)5[Tyr(Me)2-Tyr-Nh2]9-OVT (NEX254010UC, Perkin Elmer, USA). In brief, the slides were fixed in 0.1% paraformaldehyde and after washing them twice in 50 mM Tris (pH 7.4) they were exposed to the tracer buffer (50 mM tracer, 50 mM Tris, 10 mM MgCl2, 0.1% bovine serum albumin) for 60 min. Subsequently slides were washed again three times in Tris buffer 10 mM MgCl2, dipped in water and dried at room temperature overnight. On the next day, slides were exposed to Biomax MR films for 5 days (Kodak, France). The films were scanned at high resolution (1200dpi at 8-bit) using a flatbed scanner (EPSON Perfection V800, Epson Corporation, Germany). Quantification of gray value was performed in IMAGE J (NIH, Bethesda, MD, USA) with taking the mean of 3 sections per region of interest (ROI). For quantification, no adjustments were made to the images. ROIs were selected by comparison to an atlas (Paxinos and Franklin, 2019). Specific binding was determined by subtraction of tissue background sampled from a control region for each slide. Left and right regions were scored separately and pooled, if no significant hemispheric difference was found.

### OXT immunohistochemistry

To analyze OXT-positive (OXT^+^) neurons in the PVN and SON of BL6 and CD1 mice, they were transcardially perfused with 4% paraformaldehyde. Therefore, mice were deeply anaesthetized with Xylazine (2%; 0.5 mL/kg; Serumwerk Bernburg AG, Bernburg, Germany) and Ketamine (10%; 1 mL/kg; WDT, Garbsen, Germany) via an intraperitoneal injection. Subsequently, the thorax of the mice was opened and the left ventricle cannulated. After cutting the right atrium to allow efflux of blood and perfusate, animals were perfused transcardially using 0.1 M PBS and then 4% paraformaldehyde in 0.1 M PBS. Brains were collected and post-fixed overnight in 0.1 M PBS and 4% paraformaldehyde, cyro-protected in 30% sucrose in 0.1 M PBS for 2–3 days at 4°C, and snap frozen in −32°C cold n-methylbutane (Sigma Aldrich, Deisenhofen, Germany). Brains were cryo-cut in 40-μm sections and immunofluorescently stained for OXT. Therefore, free-floating sections were washed in 0.01 M PBS, blocked in 0.01 M PBS containing 0.3% Triton X-100 and 10% normal goat serum (NGS, Vector Laboratories, Burlingame, USA) for 1 h, and incubated over night at 4°C with OXT-neurophysin (PS-38, 1:500 in PBS, 0.3% Triton X-100 and 2% NGS; kindly provided by Dr. Harold Gainer, NIH, Bethesda, USA; Ben-Barak et al., 1985; Whitnall et al., 1985). After additionally washing steps, slides were incubated with the secondary antibody (Alexa Fluor^™^488 goat anti-mouse, 1:1000 in 0.01 M PBS containing 3% NGS) for 2 h at room temperature, before they were mounted with Roti^®^Mount FluorCare containing DAPI and analyzed (Leica thunder tissue imager 3D microscope, 40x objective, 5×3 pictures with 30 Z-stacks every 1 μm). For each picture, the region of interest was selected manually [paraventricular nucleus (PVN) included left and right hemispheres in one picture, while for the supraoptic nucleus (SON) left and right hemispheres were analyzed separately]. OXT^+^ cells were counted by co-localization of OXT and DAPI using ImageJ (DAPI: automated counting with analyze particle plugin; OXT: manual counting) and presented as percentage of total cell number.

### Statistical analysis

SPSS 29 (IBM) was used for statistical analysis. Data were tested for normal distribution using the Kolmogorov–Smirnov test. Parametric Student’s t-test or nonparametric Mann–Whitney U-test were performed to analyze freezing behavior during the recall of CFC, and CS-US-pairings during SFC acquisition. To analyze anxiety-like behavior in icv OXT-treated mice, a parametric one-way (factor treatment) analysis of variance (ANOVA), followed by Bonferroni *post hoc* test, was performed. A parametric two-way ANOVA was performed for analysis of HPT, bodyweight, anxiety-like behavior and locomotion in the EPM and OFT, age and strain effects of CS-US-pairings during SFC acquisition, OXT receptor binding and the percentage of OXT^+^ cells in different brain regions (factors age x strain), and of social preference behavior in OXT-treated mice (factors strain x treatment), followed by Bonferroni *post hoc* test. Responses, scores and vocalization in the FSSC, social investigation times during the SPAT, as well as during social fear extinction and recall were analyzed using mixed model ANOVA (FSSC: factor intensity x age x strain; SPAT: factor strain x stimulus; SFC without treatment: factor strain x conditioning x stimulus; SFC with treatment: factor conditioning x treatment x stimulus), followed by Bonferroni *post hoc* test, for non-social (ns1-ns3) and social (s1-s6) stimuli separately. Geisser–Greenhouse correction was applied when sphericity was violated (tested by Mauchly-test). Similar analyses (mixed model ANOVA, Bonferroni *post hoc*, Geisser–Greenhouse correction when appropriate) were performed for freezing behavior during acquisition and extinction of CFC (factor strain x stimulus). Pearson correlation was used for correlation of perception of noxious stimuli with bodyweight. Statistical significance was accepted at *p* ≤ 0.05. Statistical outliers were calculated by “mean ± 2 x standard deviation.” Significant *p*-values are stated in the text. Detailed reports for all analyses are available in Supplementary Tables, to avoid excessive text passages. Graphs were plotted using Prism 9 (GraphPad).

## Results

### Strain differences in anxiety-related behavior, locomotor activity and social preference behavior in adolescent and adult BL6 and CD1 mice

**Figure 1:**
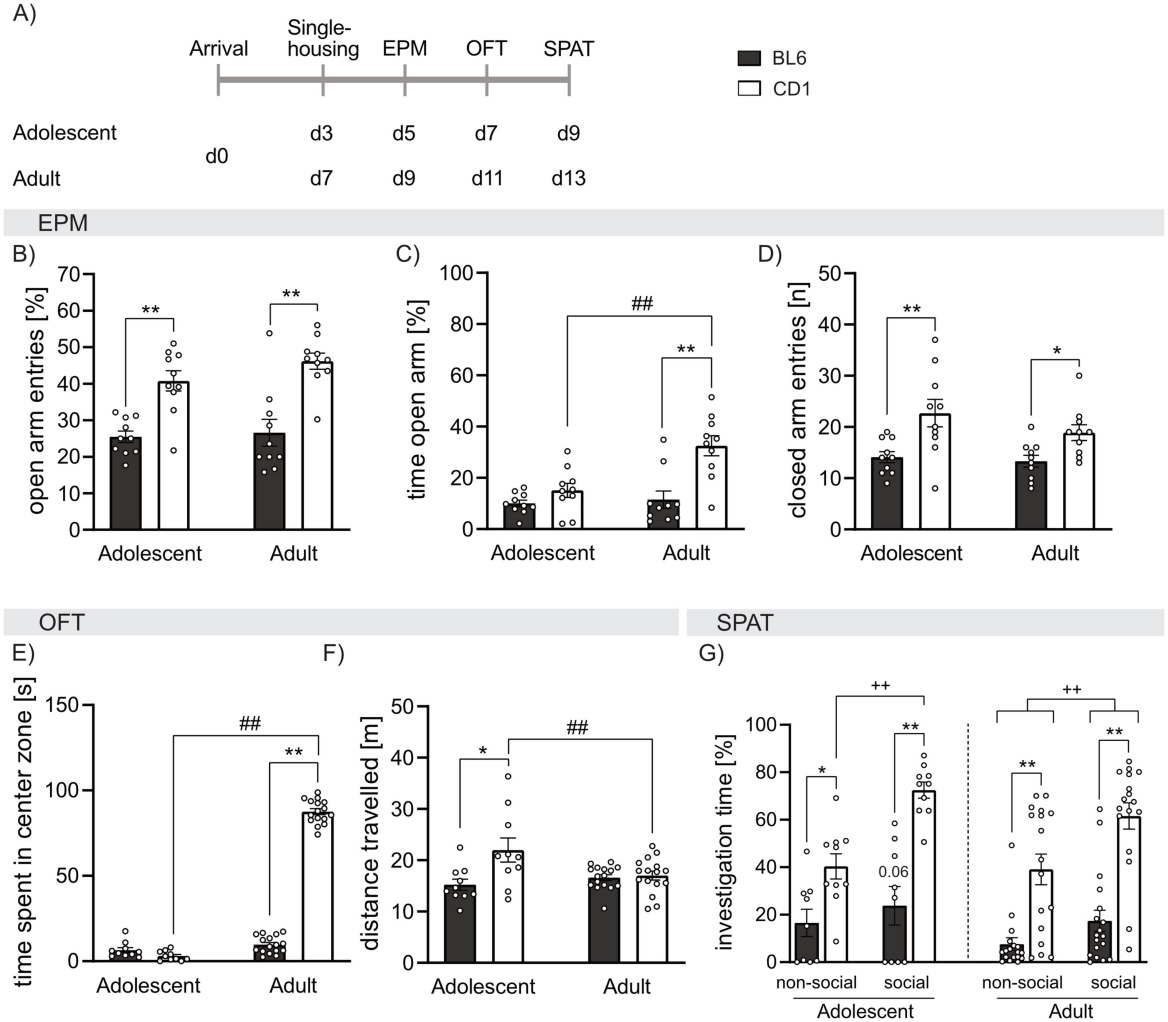
Strain differences in anxiety-related behavior, locomotion, and social preference behavior in adolescent and adult male BL6 and CD1 mice. (A) Schematic representation of the experimental time plan for assessing anxiety-related behavior, locomotion, and social preference using the elevated plus-maze (EPM), open field test (OFT) and social preference/avoidance test (SPAT). (B) Percent of open arm entries of the EPM of adolescent and adult BL6 and CD1 mice. (C) Percent of time spent on the open arm of the EPM of adolescent and adult BL6 and CD1 mice. (D) Number of closed arm entries on the EPM adolescent and adult BL6 and CD1 mice. (E) Time spent in the center zone (in sec) in the OFT of adolescent and adult BL6 and CD1 mice. (F) Distance traveled in the OFT arena of adolescent and adult BL6 and CD1 mice. (G) Percent of investigation time of the non-social and social stimulus of adolescent and adult BL6 and CD1 mice during the SPAT. EPM: *n* = 10/group; OFT: *n* = 10/adolescent group, *n* = 16/adult group; SPAT: *n* = 10/adolescent group, *n* = 17–18/adult group. *p < 0.05, **p < 0.01 BL6 vs. CD1; ##*p* > 0.01 adult vs. adolescent; ++*p* < 0.01 investigation of non-social vs. social stimulus, *p* = 0.06 non-social vs. social investigation in adolescent BL6 mice. Data represent mean ± SEM. Each dot represents an individual mouse. For detailed statistics (see Supplementary Table S1).

To assess strain- and age-dependent differences in general anxiety-related behavior and locomotion, adolescent and adult BL6 and CD1 mice were exposed to the EPM and OFT. Both, adolescent and adult BL6 mice showed lower percentage of open arm entries compared to respective CD1 mice (*p* < 0.001; Figure 1B), but only adult BL6 mice spent less time in the open arms compared to CD1 mice (*p* < 0.001; Figure 1C). Moreover, BL6 mice of both age groups show equal amounts of open arm entries and time spent on the open arm. Contrarywise, adult CD1 mice spent more time on the open arm compared to adolescents of the same strain (*p* < 0.001, Figure 1B), while adolescent and adult CD1 mice entered the open arm with the same the same frequency (Figure 1B). These data suggest higher anxiety-related behavior and less exploratory drive especially in adult BL6 animals. At both ages, the high anxiety-related behavior of BL6 mice was associated with lower locomotor activity depicted by decreased closed arm entries on the EPM compared to CD1 mice (adolescents: *p* < 0.001; adults: *p* = 0.029; Figure 1D). No age-dependent effects on closed arm entries have been found within BL6 and CD1 mice.

In support of the observed differences in anxiety-related behavior and locomotion on the EPM, adult, but not adolescent, BL6 mice spent less time in the center zone of the OF compared to adult CD1 mice (*p* < 0.001, Figure 1E). Moreover, adolescent, but not adult, BL6 mice showed reduced locomotor activity in the OFT compared to respective CD1 mice (*p* < 0.001 versus CD1, Figure 1F). Analyzing age effects, within the BL6 strain, adolescent and adult mice did not differ in the time spent in the center zone and distance traveled, whereas adult CD1 spent increased time in the center (*p* < 0.001), but decreased locomotion (*p* = 0.006), compared to adolescent CD1 males.

To characterize social preference behavior, adolescent and adult mice were exposed to the SPAT (Figure 1G). Independent of their age, BL6 mice showed reduced investigation time toward both presented non-social (ns) and social (s) stimuli compared to respective CD1 mice (adolescent: ns *p* = 0.047, s *p* = 0.001; adult: ns *p* < 0.001, s *p* < 0.001). However, mice of both strains spent significantly more time in contact with the social stimulus compared to the non-social stimulus reflecting normal social preference behavior (adolescent: BL6 *p* = 0.066, CD1 *p* < 0.001; adult: BL6 *p* = 0.020, CD1 *p* < 0.001). Analyzing age-effects, we further showed that adolescent BL6 mice spent more time investigating the non-social stimulus (*p* = 0.039), but not the social stimulus, compared to adult BL6, whereas in CD1 mice, no age-dependent effect of investigation time was found. This further highlights the anxious phenotype of BL6 mice, which seems to exacerbate in adulthood.

### Strain differences in cued and social fear conditioning in adolescent and adult BL6 and CD1 mice

To compare fear-related behavior, adolescent and adult BL6 and CD1 mice were subjected to the CFC and SFC paradigms.

**Figure 2:**
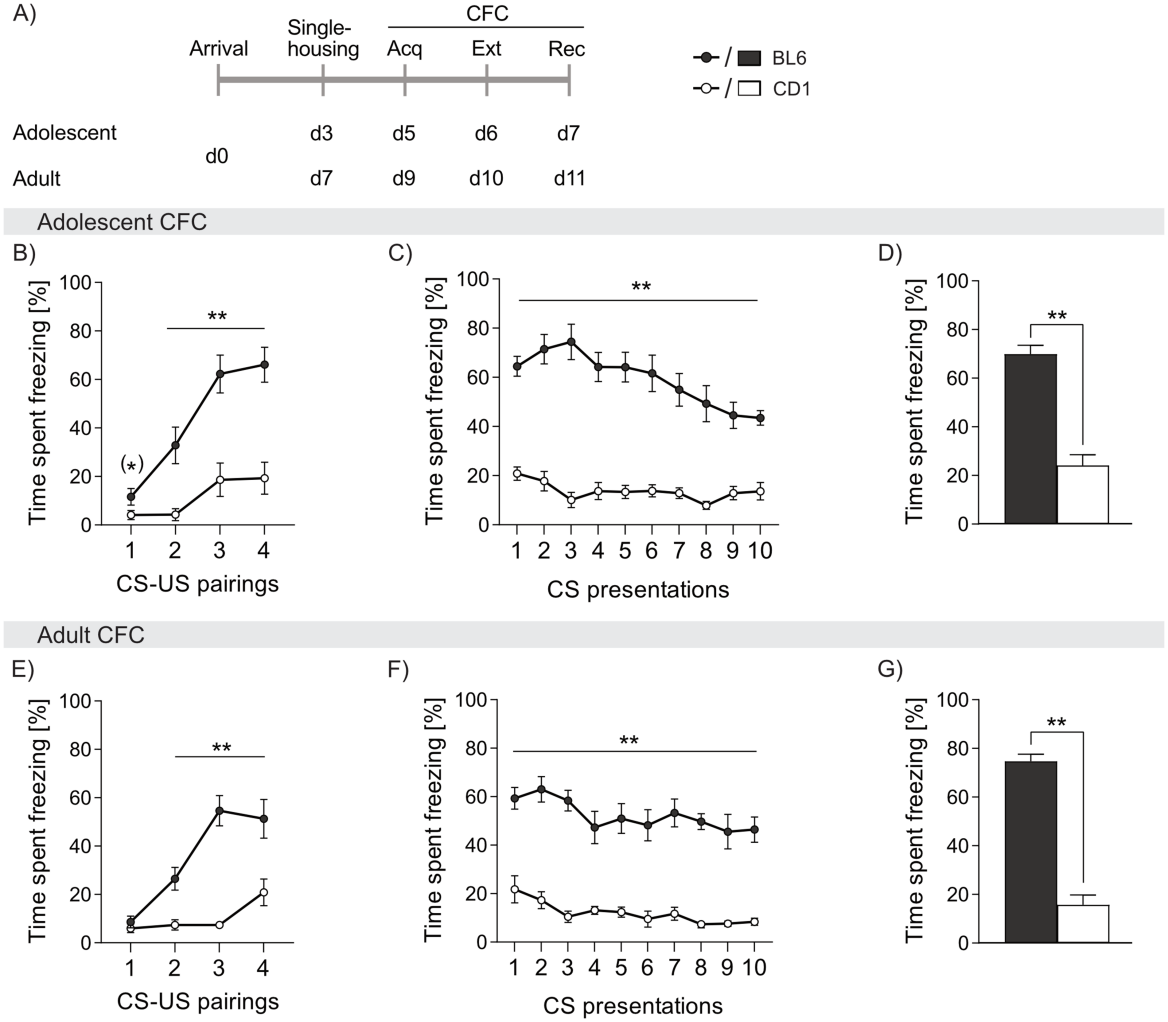
Strain differences in freezing behavior in response to cued fear conditioning (CFC) in adolescent and adult male BL6 and CD1 mice. **(A)** Schematic representation of the experimental time plan for assessing acquisition, extinction and recall of cued fear. Percentage of time spent freezing in **(B-D)** adolescent and **(E-G)** adult BL6 and CD1 mice during **(B, E)** acquisition, **(C, F)** extinction and **(D, G)** recall of cued fear. *n* = 10/group. **p < 0.01, (*) *p* < 0.07 BL6 vs. CD1. Data represent mean ± SEM. For detailed statistics (see Supplementary Table S2).

During acquisition of cued fear, both adolescent and adult BL6 mice showed three to four-fold higher freezing levels during presentation of the CS (adolescent: cs2 *p* = 0.002, cs3 and cs4 *p* < 0.001; adult: cs2 *p* = 0.002, cs3 *p* < 0.001, cs4 *p* < 0.006, Figures 2B,E). Throughout cued fear extinction, BL6 mice exhibited an increased percentage of freezing, irrespective of age (adolescent: cs1-10 *p* < 0.001; adult: cs1-10 *p* < 0.001 vs. CD1, Figures 2C,F). Although freezing responses slowly declined in BL6 mice during fear extinction, they remained at higher level compared to CD1 mice. Consequently, during recall of cued fear, BL6 mice displayed higher percentages of freezing compared to CD1, independent of age (adolescent: *p* < 0.001; adult: *p* < 0.001, Figures 2D,G). Thus, both adolescent and adult BL6 animals show an exaggerated cued fear response and incomplete fear extinction compared to CD1 males. During acquisition, extinction and recall of cued fear, no age-dependent differences were found in BL6 and CD1 mice (separate statistics, see Supplementary Table S2).

**Figure 3:**
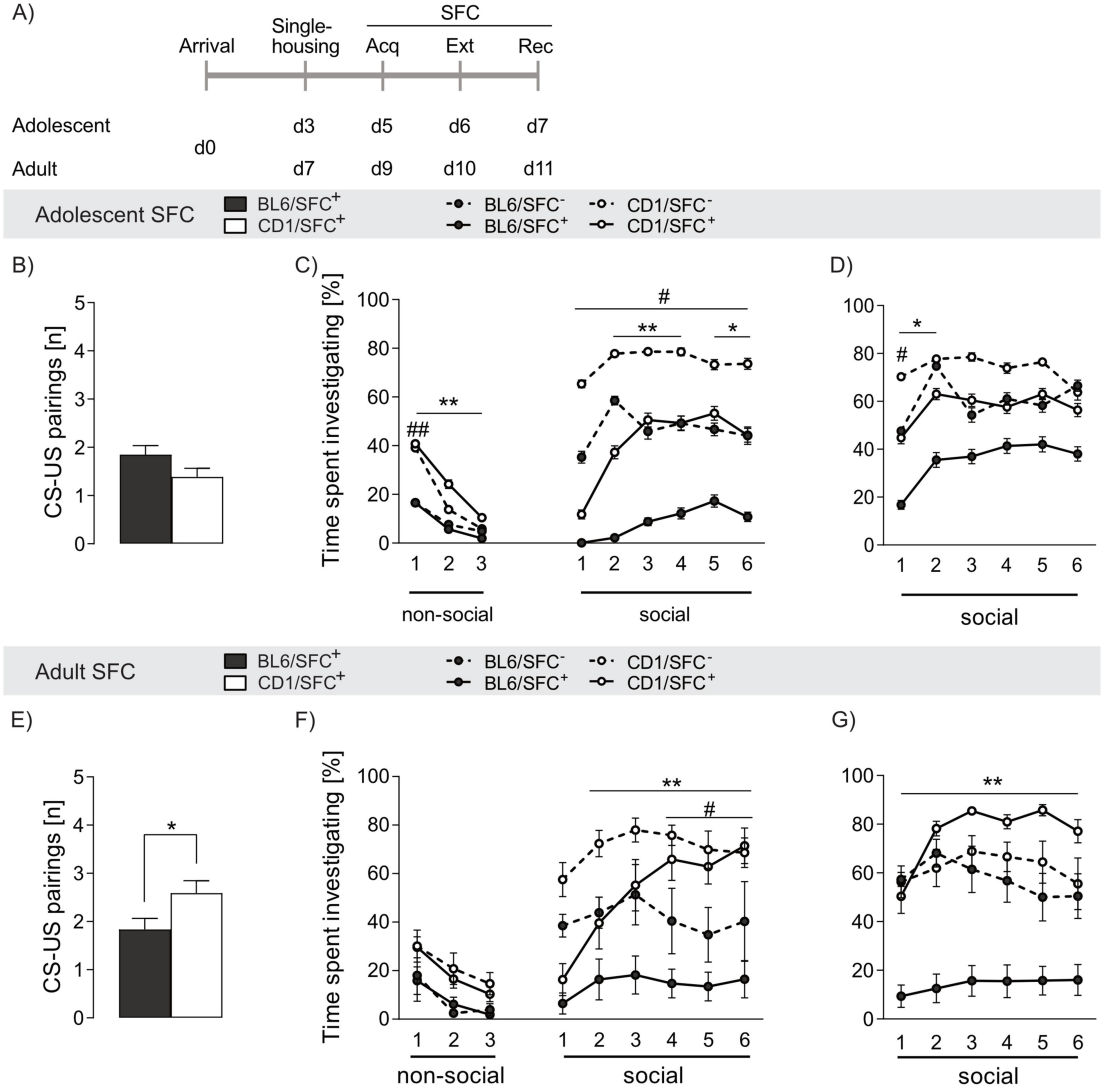
Strain differences in social fear acquisition and investigation time in response to social fear conditioning (SFC) in adolescent and adult male BL6 and CD1 mice. (A) Schematic representation of the experimental time plan for assessing acquisition, extinction and recall of social fear. (B,E) CS-US pairings during social fear acquisition of (B) adolescent and (E) adult BL6 and CD1 mice. (C) Percentage of time spent investigating non-social and social stimuli during social fear extinction and (D) recall of adolescent BL6 and CD1 mice. (F) Percentage of time spent investigating non-social and social stimuli during social fear extinction and (G) recall of adult BL6 and CD1 mice. Data represent mean ± SEM. *n* = 11-18/group. *p < 0.05, **p < 0.01 BL6/SFC^+^ vs. CD1/SFC^+^; #*p* < 0.05 ##*p* < 0.01 BL6/SFC^−^ vs CD1/SFC^−^. For detailed statistics (see Supplementary Table S3).

Adolescent SFC^+^ mice of both strains required a similar number of CS-US pairings during acquisition of social fear to induce social avoidance (*p* = 0.101, Figure 3B), whereas a lower number of CS-US pairings was needed in adult SFC^+^ BL6 (*p* = 0.027 vs. CD1, Figure 3E). Moreover, adolescent CD1, but not BL6, mice required less CS-US pairings compared to adult conspecifics (*p* = 0.028), suggesting a higher threshold for induction of a social trauma in adult CD1 mice (see Supplementary Table S3).

During social fear extinction training, no differences in exploring the non-social stimuli was found between SFC^+^ and SFC^−^ mice independent of strain and age (Figures 3C,F), confirming that SFC does not alter general anxiety-related and exploratory behavior (Toth et al., 2012b).

SFC^+^ mice of both strains and age groups expressed social fear as reflected by low investigation of the presented conspecific during presentation of the first social stimulus (Figures 3C,F). In line with decreased social interaction shown in the SPAT (Figure 1G), adolescent and adult BL6/SFC^−^ animals displayed lower social investigation compared to respective CD1/SFC^−^ (adolescent: s1 *p* = 0.001, s2-6 p ≤ 0.05, adult: s4-6 p ≤ 0.05). Further, whereas CD1/SFC^+^ mice gradually increased social interaction during consecutive presentation of the six social stimuli, BL6/SFC^+^ remained at low levels of social investigation times irrespective of age (adolescent: s2-3 *p* < 0.001, s4 *p* = 0.007, s5-6 *p* < 0.05, adult: s2-6 *p* < 0.001) compared to CD1/SFC^+^ mice. Unconditioned (SFC^−^) adolescent BL6 mice spent more times investigating the first social stimulus (*p* = 0.008) compared to adult BL6 mice, whereas no age-related differences were found in SFC^+^ BL6 mice. In opposite, no age-dependent differences were found in SFC^−^ CD1 mice, whereas in SFC^+^ CD1 males adult animals spent more time investigating the s4 (*p* = 0.025) and s6 (*p* = 0.005) social stimulus, compared to respective adolescents. These data indicate an exacerbation of trauma-induced fear throughout development of BL6 mice, while CD1 mice seem to have a more resilient fear-related behavior in adulthood compared to adolescence.

Analyzing age-effects, adolescent BL6/SFC^−^ (ns1 *p* = 0.002) and BL6/SFC^+^ (ns1-3 *p* < 0.01) animals showed overall lower investigation of the non-social stimuli compared to respective CD1 mice, confirming their higher fear level. Moreover, no age-dependent effect on investigation of the non-social stimuli was found in unconditioned and conditioned BL6 and CD1 mice, respectively.

During social fear recall, both adolescent and adult BL6/SFC^+^ mice displayed lower social investigation (adolescent: s1-2 *p* < 0.05, adult: s1-6 *p* < 0.001 vs. CD1/SFC^+^, Figures 3D,G), indicating incomplete extinction of social fear and enhanced social fear memory in BL6 mice. Interestingly, SFC^+^, but not SFC^−^, adolescent BL6 mice showed higher investigation times compared to their adult conspecifics (s2, s4, s5 *p* < 0.05), indicating even exacerbated social fear responses during adulthood in BL6 mice.

### Strain differences in perception of electric foot shocks and thermally induced pain in adolescent and adult BL6 and CD1 mice

**Figure 4:**
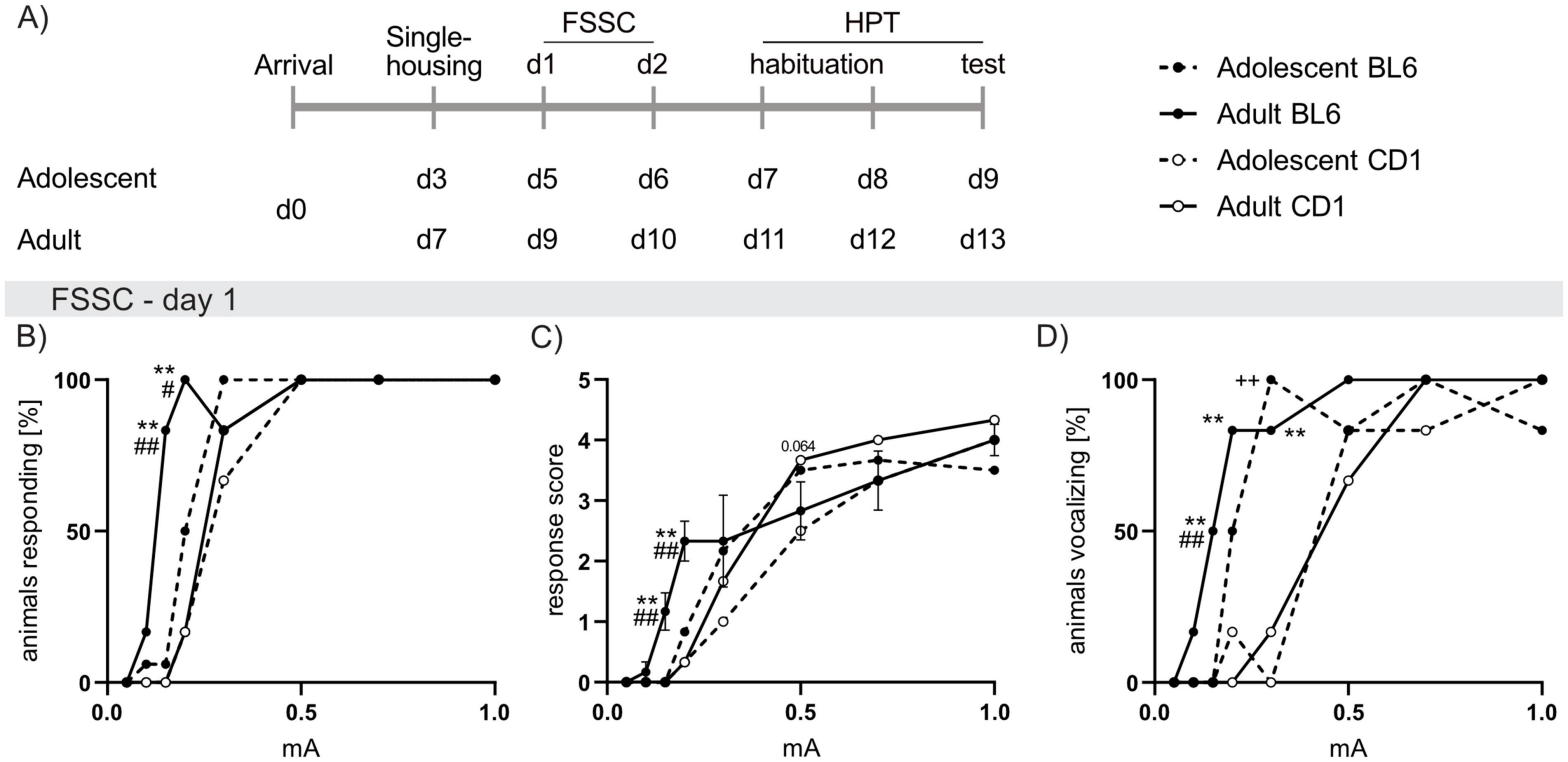
Strain differences in responses to foot shock exposure of increasing intensities in adolescent and adult male BL6 and CD1 mice. (A) Schematic representation of the experimental time plan for perception of electric (Foot shock sensitivity comparison; FSSC) and thermal (Hargreave’s plantar test; HPT) noxious stimuli. (B) Percent of adolescent and adult BL6 and CD1 mice responding to increasing foot shock intensities (0.05 mA, 0.1 mA, 0.15 mA, 0.2 mA, 0.3 mA, 0.5 mA, 0.7 mA, 1.0 mA) during the first day of FSSC. (C) Response score on the first day of FSSC of adolescent and adult BL6 and CD1 mice. (D) Percent of adolescent and adult BL6 and CD1 mice vocalizing during the first day of FSSC. *n* = 5/group. +*p* < 0.05, ++*p* < 0.01 adolescent BL6 vs. CD1; *p < 0.05, **p < 0.01 adult BL6 vs. CD1; #*p* < 0.05, ##*p* < 0.01 adult vs. adolescent BL6. Data represent mean ± SEM. SEM is not visible in graph whenever the value was too small. For detailed statistics (see Supplementary Table S4).

FSSC analysis revealed revealed increased responsiveness in adult and adolescent BL6 compared to respective CD1 mice. This was reflected by increased percentage of adult BL6 mice responding to low foot shock intensities (0.15 mA: *p* < 0.001; 0.2 mA: *p* = 0.002; Figure 4B) in conjunction with heightened response scores (0.25 mA and 0.2 mA *p* < 0.001 versus CD1, Figure 4C). Additionally, a higher percentage of adolescent (0.3 mA: *p* < 0.001 vs. CD1; Figure 4D) and adult (0.15 mA: *p* = 0.005, 0.2 mA: *p* = 0.002, 0.3 mA: *p* < 0.001 versus CD1) BL6 mice vocalized during foot shock presentations. Further, a higher percentage of adult BL6 mice responded to 0.15 mA (*p* < 0.001; Figure 4B) and 0.2 mA (*p* = 0.042) with increased response scores (0.15 mA *p* < 0.001; 0.2 mA *p* = 0.007; Figure 4C) when compared to adolescents of the same strain. On the second day of the FSSC, the increased foot shock sensitivity of adolescent and adult BL6 mice was only found in the percentage of animals vocalizing in BL6 compared to CD1 mice (Supplementary Figures S1A–C).

In the HPT, during adolescence no difference in the latency to withdraw the paw was found between strains. Confirming the results of FSSC, adult BL6 mice responded with a lower paw withdrawal latency (*p* = 0.021 vs. CD1; Supplementary Figure S1D). Moreover, adolescent mice of both strains showed lower paw withdrawal latencies when compared to the adults of the same strain (BL6: *p* = 0.048, CD1: *p* < 0.001).

At the day of FSSC and HPT, BL6 males of both ages showed decreased bodyweight compared to respective CD1 mice (FSSC: adolescent: *p* = 0.015; adult: *p* < 0.001; HPT: adolescent and adult: *p* < 0.001; Supplementary Figure S1F). Moreover, adolescent CD1 mice showed decreased bodyweight compared to adult CD1 mice, whereas in BL6 mice no age-specific effect on bodyweight was found (*p* < 0.001). Interestingly, the paw withdrawal latency positively correlated with bodyweight, bodyweight, when analyzed irrespective of strain and age (*p* = 0.011; Supplementary Figure S1E). No correlation was found between the bodyweight and the response score to foot shocks during FSSC (Supplementary Table S7).

### Strain differences in the OXT system in naive adolescent and adult BL6 and CD1 mice

**Figure 5:**
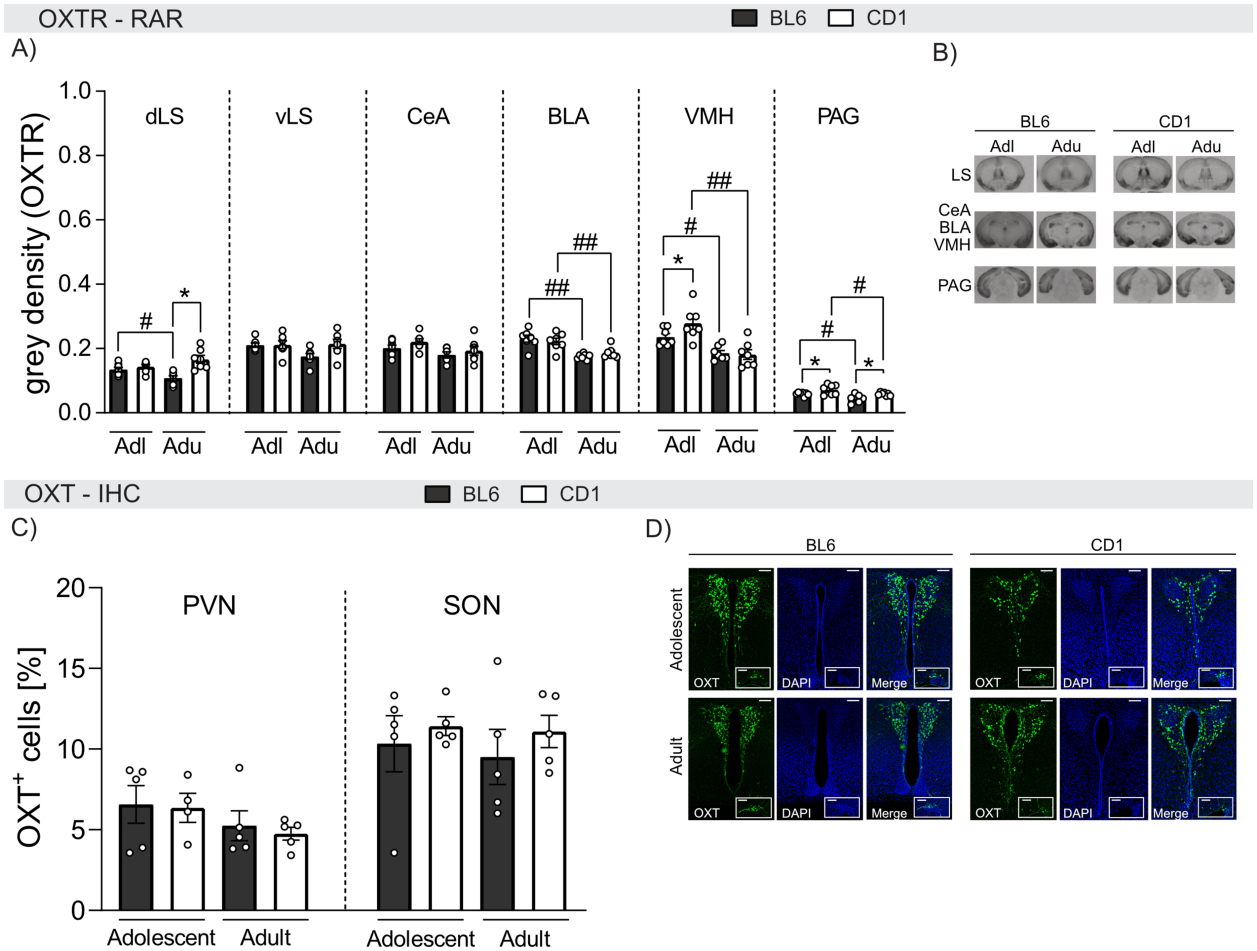
Strain differences in OXTR binding and quantity of OXT-positive cells in selected brain regions of adolescent and adult male BL6 and CD1 mice. (A) OXTR binding in the dorsal lateral septum (dLS), ventral lateral septum (vLS), central amygdala (CeA), basolateral amygdala (BLA), ventromedial nucleus of the hypothalamus (VMH), and periaqueductal gray (PAG) of adolescent (Adl) and adult (Adu) BL6 and CD1 male mice. (B) Representative images of the analyzed brain regions using OXTR autoradiography including all groups. (C) OXT^+^ cells in the paraventricular nucleus (PVN) and the supraoptic nucleus (SON) of adolescent and adult male BL6 and CD1 mice. (D) Representative images of PVN and SON of all analyzed groups. RAR *n* = 6–7/group; IHC *n* = 3–5/group. Data represent mean ± SEM.*p < 0.05 BL6 vs. CD1; #*p* < 0.05, ##*p* < 0.01 adolescent vs. adult. For detailed statistics (see Supplementary Table S5).

A putative mediator of the observed behavioral differences between BL6 and CD1 mice is CD1 mice is oxytocin. Hence, we compared OXTR binding by means of OXTR autoradiography in adolescent and adult naïve male BL6 and CD1 mice (Figures 5A,B).

Within the dorsal LS (dLS), OXTR binding was age^−^ and strain-dependent. In detail, OXTR binding was found to be higher in adolescent compared to adult BL6 animals (*p* = 0.038), and lower in adult BL6 compared to adult CD1 mice (*p* < 0.001). However, no differences were found within the ventral LS (vLS).

In the BLA and VMH, OXTR binding was found to be age-dependent in both BL6 and CD1 mice with higher binding in adolescent compared to adult mice (BLA: BL6: *p* < 0.001, CD1: *p* = 0.006; VMH: BL6: *p* = 0.017, CD1: *p* < 0.001). Strain differences were only found within the VMH of adolescent mice, with lower OXTR binding in BL6 compared to CD1 mice (*p* = 0.020). Similarly, elevated OXTR binding was found within the PAG of adolescent compared to adult mice independent of the strain (BL6: *p* = 0.025, CD1: *p* = 0.027). Also, OXTR binding was lower in both adolescent (*p* = 0.032) and adult (*p* = 0.029) BL6 compared to respective CD1 mice. In the CeA, OXTR binding did not differ between strains or ages.

In summary, OXTR binding in the BLA, VMH, and PAG decreased in adulthood in both strains, but was overall lower in BL6 compared to CD1 mice. These differences might contribute to the observed behavioral strain differences.

Finally, we quantified OXT^+^ cells in the PVN and SON of adolescent and adult BL6 and CD1 mice (Figures 5C,D). However, quantities did not differ between age groups or strains in either PVN or SON (Figure 5C).

### Strain differences in the effects of icv OXT on anxiety-related and social preference behaviors in adult BL6 and CD1 mice

**Figure 6:**
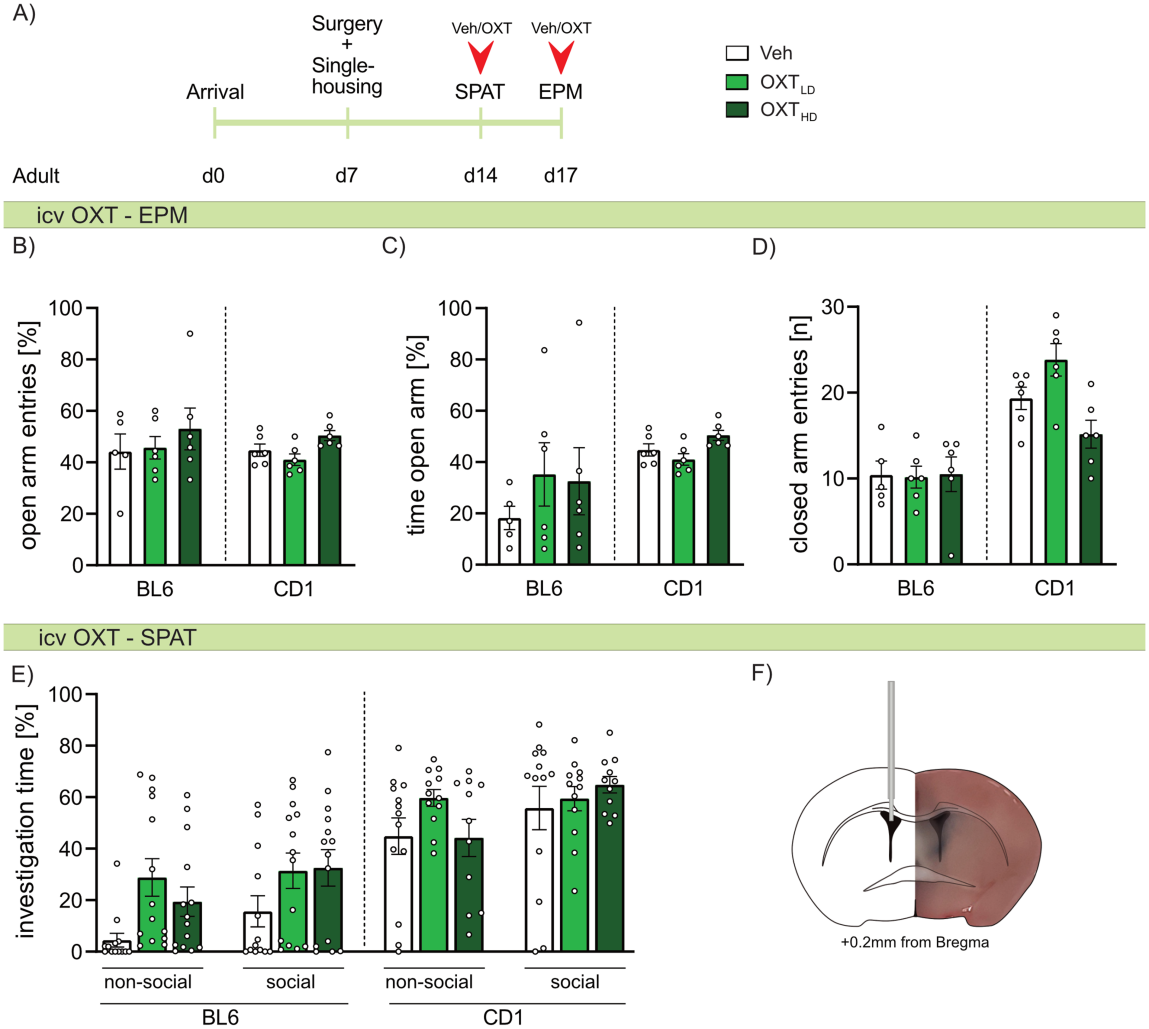
Effect of intracerebroventricular (icv) oxytocin (OXT) on anxiety-related behavior, locomotion, and social preference behavior in adult male BL6 and CD1 mice. (A) Schematic representation of the experimental time plan for assessing the effect of icv OXT infusion on anxiety-related behavior, locomotion and social preference using the elevated plus-maze (EPM) and social preference/avoidance test (SPAT). (B) Percent open arm entries, (C) percent of time spent on the open arm, and (D) number of closed arm entries on the EPM, as well as (E) investigation time of a non-social and social stimulus during SPAT of adult BL6 and CD1 mice infused with either vehicle (Veh), a low dose (OXT_LD_ = 0.1 μg/2 μL) or a high dose (OXT_HD_ = 0.5 μg/2 μL) of OXT. (F) Representative image of icv cannula placement. *n* = 11-14/group. Data represent mean ± SEM. For detailed statistics (see Supplementary Table S6).

As we found strain-specific differences in OXTR binding, we further evaluated, whether icv OXT can at least partially ameliorate the differences in anxiety-related and social behavior between adult BL6 and CD1 mice. Hence, adult mice of both strains were infused with Veh, or a low or high dose of OXT 20 min prior to behavioral assessment. On the EPM, neither the high nor low dose of OXT affected anxiety-related behavior (Figures 6B,C) or locomotor activity (Figure 6D) in either strain. Similarly, high or low doses of icv OXT did not alter the investigation of the non-social or social stimulus presented in the SPAT in either strain (Figure 6E). Further, extinction of social fear in adult BL6 mice was not changed by icv OXT infusion (Supplementary Figure S2).

## Discussion

The selection of the mouse strain for any given biomedical research project is of high relevance for the outcome and interpretation of the results. When investigating various aspects of emotional behavior, especially in the context of susceptibility to stress-induced maladaptations, either a vulnerable or resilient mouse strain might be of advantage, depending on the surveyed research question. Similarly, studying social behavior, the choice of a socially motivated, highly aggressive or non-aggressive mouse strain seems to be essential. The data of the present study supports the necessity of a sophisticated selection of a mouse strain, as we show fundamental differences in anxiety- and fear-related, as well as social behavior between male BL6 and CD1 mice. Interestingly, some of these behavioral differences can already be detected in adolescence. Moreover, we demonstrated that the brain OXT system differs between the two mouse lines with reduced OXTR binding especially in the dLS, VMH, and PAG, indicating that such differences might contribute to the observed behavioral phenotypes. However, icv OXT treatment in adult BL6 mice did not reverse their susceptible phenotype, as it did neither alter anxiety-related behavior, nor social preference or extinction of social fear.

Assessed in the EPM and OFT, we found increased anxiety in adult BL6 compared to CD1 mice (Figure 1), thus confirming recent results in the novel open space (Michalikova et al., 2010) and rat exposure test (Yang et al., 2004). In BL6 mice, anxiety levels did not significantly differ in adolescence, suggesting an enduring highly anxious phenotype throughout development. Indeed, whereas in CD1 mice anxiety levels were found to decrease in adulthood compared to adolescence, reflected by, for example, increased time spent in the open arms or center zone of the EPM and OFT, respectively, anxiety levels remained unchanged in adolescent and adult BL6 mice.

In line, both, adolescent and adult BL6 mice showed reduced locomotor activity in the EPM and OFT (Figure 1), which has repeatedly been associated with hyper-anxiety in rodents (Gryksa et al., 2023; Ramos et al., 1997; Tang and Sanford, 2005), further highlighting the high susceptibility of BL6 mice.

Both, adult BL6 and CD1 mice display natural social preference, as tested in the SPAT (Figure 1), indicated by a higher investigation time of the social compared to the non-social stimulus. However, adolescent and adult BL6 animals displayed lower levels of social interaction in the SPAT, reflected by the lower time spent investigating the conspecific compared to respective CD1 mice, which renders the BL6 strain less suitable to study mechanisms underlying social motivation. These differences in social behavior were also found between female BL6J and CD1 mice (Ramsey et al., 2021) during adolescence, only CD1 but not BL6 mice showed social preference behavior, indicating important age- and strain-specific differences in social motivation. In support, differences between young and aged BL6J mice in anxiety-like behavior, locomotion, as well as social behavior have already been reported (Shoji et al., 2016; Shoji and Miyakawa, 2019). Hence, our study further highlights the critical period of adolescence in the development of psychopathologies associated with social deficits, as well as a high level of individual susceptibility (Tillfors, 2004; Wright et al., 2020). Interestingly and in support of our findings in EPM and OFT, adolescent BL6 mice spent more time investigating the non-social stimulus, compared to adult BL6, which further highlights our hypothesis of an exacerbated anxious phenotype of adult BL6 mice.

We further compared non-social (CFC; Figure 2) and social (SFC; Figure 3) fear responses in adolescent and adult male BL6 and CD1 mice. During CFC, BL6 mice, independent of age, exhibited increased freezing from the second CS-US pairing on compared to respective CD1 mice. This resulted in impaired cued fear extinction and persistence of the conditioned fear even during cued fear recall on day 3 in BL6 mice independent of their age. These findings are supported by higher freezing behavior in BL6 mice compared to other strains in the CFC paradigm (e.g., DBA/2 and BALB/c mice) (Chen et al., 1996; Gerlai, 1998; Stiedl et al., 1999). Further, when comparing inbred strains (C57BL/6J, 129S1/SvImJ, A/J, BALB/cByJ, C3H/HeJ, and others) BL6J mice showed a more pronounced startle response to the tone (CS), even without previous CS-US coupling (Bolivar et al., 2001). Interestingly, neither BL6 nor CD1 mice showed age-dependent differences during CFC acquisition, extinction, and recall, demonstrating stable non-social fear learning and memory capabilities throughout development.

Robust strain differences were also revealed during social fear acquisition and extinction, both in adolescent and adult mice (Figure 3). During acquisition of social fear, adult, but not adolescent BL6 mice required less CS-US pairings to avoid further investigation of the conspecific, which indicates a higher susceptibility to trauma-induced social fear in adult BL6 compared to same-aged CD1 mice. Similar to cued fear extinction, both adolescent and adult SFC^+^ BL6 mice displayed an impaired social fear extinction, demonstrated by overall low social investigation times throughout consecutive presentation of the six social stimuli. This is in contrast to SFC^+^ CD1 mice of both ages, which show a gradual increase in investigation times throughout consecutive exposure to 6 different social stimuli indicating successful extinction of social fear. Interestingly, we found age-dependent differences during SFC. Here, BL6 mice were more vulnerable in adulthood compared to adolescent conspecifics, whereas CD1 mice showed incomplete extinction during adolescence only, illustrated by decreased social investigation of SFC^+^ compared to SFC^‒^ mice.

These data indicate an exacerbation of social trauma-induced fear throughout development of BL6 mice, whereas CD1 mice seem to develop a more resilient fear-related behavior during adulthood compared to adolescence. Hence, we provide evidence that BL6 mice are highly vulnerable to trauma-induced non-social and social fear both in adolescence and adulthood, whereas CD1 mice can be characterized as being rather resilient and able to successfully extinguish non-social and social fear. Thus, BL6 mice might be a suitable model to study exposure therapy-resistant fear.

Additionally, we compared the response of adolescent and adult BL6 and CD1 males to foot shocks of increasing intensities (FSSC; Figure 4) and a thermal pain stimulus (HPT; Supplementary Figure S1). Here, BL6 mice showed a heightened response to low foot shock intensities and a decreased paw withdrawal time in response to a heat stimulus, which, in line to the found differences in anxiety and fear-related behavior, seems to exaggerate during adulthood. On the second day of FSSC, especially adult BL6 mice showed increased vocalization at mild foot shock intensities, whereas similar response scores of both strains and age groups were found, revealing a converging response of both strains (Supplementary Figure S1).

Likewise, during the HPT, adult but not adolescent BL6 mice showed decreased paw withdrawal latencies compared to CD1 mice, which positively correlated to the animal’s bodyweight, revealing increased pain perception in response to a thermal stimulus. These results are in line to literature showing strain differences in pain perception (Smith, 2019), which might be based on differences in bodyweight (Domínguez-Oliva et al., 2022). However, the HPT is not fully accurate to compare the perception of pain between BL6 and CD1 mice as skin pigmentation substantially influences infrared beam-induced heat perception, resulting in a physiologically increased paw withdrawal latency in albino CD1 mice compared to highly pigmented BL6 mice.

Hence, the perception of noxious stimuli such as electric foot shocks and heat seem to be not exclusively influenced by the bodyweight, since a positive correlation between bodyweight and HPT, but not FSSC, was found. Thus, especially foot shock perception seems to be dependent on other factors, e.g., age and emotional perception.

Substantial sex differences exist in socio-emotional behaviors (Bangasser and Cuarenta, 2021). Although it was not the intention to add sex differences to the experimental design of this study, it is likely that also female CD1 and BL6 mice differ in innate behaviors. Indeed, female Bl6 and BL6J mice were described to be more anxious in the light–dark box (Medina-Saldivar et al., 2024), less socially motivated (Ramsey et al., 2021), and showed higher maternal aggression (Gryksa et al., 2020) compared to CD1 mice.

To date, the neurobiological mechanisms underlying the observed differences in anxiety- and fear-related as well as social behavior between distinct mouse strains remain elusive. However, differences in the brain OXT system with its anxiolytic, pro-social, stress-buffering, and anti-inflammatory properties (Froemke and Young, 2021; Jurek and Neumann, 2018; Menon and Neumann, 2023) may contribute to the observed distinct behaviors. Indeed, we have identified strain- and age-dependent differences of OXTR binding in various brain regions that are critically involved in anxiety-like behavior, conditioned fear and social behavior (Figure 5; Brandão et al., 2008; Calhoon and Tye, 2015; Menon et al., 2022; Zhao et al., 2009; Zoicas et al., 2014). Compared to male CD1 mice, lower levels of OXTR binding were identified within the VMH (adolescents), dLS (adults), and PAG (adolescents and adults) of BL6. Whereas the LS is essentially modulating social behavior including social fear extinction (Menon et al., 2022), the VMH is particularly involved in anxiety and fear responses (Zhao et al., 2009) and the PAG in fear, flight and freezing responses (Brandão et al., 2008; Tovote et al., 2015; Tovote et al., 2016; Zhao et al., 2009), leading to the hypothesis that differences in local OXTR binding might contribute to the observed strain and age differences in socio-emotional behavior (see Figures 1–4).

Importantly, developmental alterations in OXTR binding were found in both BL6 and CD1 mice. In general, OXTR binding was lower in adult compared to adolescent mice in the BLA, VMH, and PAG. In support, OXTR binding has previously been found to be reduced within the dLS and PVN of adult compared to juvenile male and female BL6J mice (Olazábal and Alsina-Llanes, 2016). Generally, OXT signaling during development has been shown to affect physiology and behavior during adulthood (Vaidyanathan and Hammock, 2017). However, when the amount of OXT^+^ cells within the PVN and SON were analyzed using IHC (Figure 5), no age- or strain-dependent effect was found, indicating that there is no general difference of OXT-positive neurons, within the developmental stages and strains.

Based on our behavioral studies and the altered OXT system in BL6 mice, we consequently investigated, whether acute icv OXT infusion exerts beneficial effects on anxiety-like and social behavior, especially in adult BL6 mice (Figure 6). In our study, neither low (OXT_LD_ 0.1 μg/2 μL) nor high (OXT_HD_ 0.5 μg/2 μL) OXT reduced anxiety-like behavior or affected social fear extinction in adult BL6 and CD1 mice. This contrasts several previous studies demonstrating anxiolytic and pro-social properties of OXT (Froemke and Young, 2021; Jurek and Neumann, 2018; Menon and Neumann, 2023). However, a robust anxiolytic effect of OXT has only been described in rats so far, specifically after OXT has been infused locally into the PVN or amygdala, but not after icv infusion. Moreover, a prerequisite for detecting an anxiolytic effect of OXT applied either locally or icv is the exposure of the experimental animal (rat or mouse) to mild pre-stress (Bale et al., 2001; Blume et al., 2008; Ring et al., 2006; Winter et al., 2021; Yoshida et al., 2009). Specifically, an anxiolytic effect of icv OXT was only detected in mice, which were pre-stressed either by anesthesia and surgery for icv infusion or by ip injection 30 min prior to icv infusion and behavioral testing (Ring et al., 2006; Yoshida et al., 2009). In the present study, we expected an anxiolytic effect of icv OXT especially in BL6 mice due to their general high stress susceptibility; this expectation, however, was not fulfilled. Thus, pre-exposure to a mild stressor prior to icv OXT infusion and subsequent anxiety testing seems to be crucial in mice independent of their innate phenotype. This is in accordance with the social salience hypothesis of OXT, which implies that the effects of OXT may depend on the internal emotional state of an individual, which is shifted by previous stressor exposure. Thus, OXT may shift the salience of an emotional context, rather than acting unidirectional on any kind of behavior (e.g., anxiety) (Shamay-Tsoory and Abu-Akel, 2016). Hence, in unstressed conditions, an anxiolytic effect of OXT might not be visible.

With respect to social fear, although icv OXT treatment was originally described to reduce social fear expression in CD1 males (Zoicas et al., 2014), a robust effect on social fear expression was only detectable after OXT was locally infused into the LS (Bludau et al., 2024; Menon et al., 2018; Zoicas et al., 2014). However, alterations in the emotional state of mice as a consequence of surgical interference (implantation of the icv guide cannula 5 days prior to behavioral testing) seem to be more profound in stress vulnerable BL6 compared to resilient CD1 mice. Indeed, in both Veh- and OXT-treated, SFC^+^ and SFC^–^ BL6 mice, we observed overall low social investigation times throughout social fear extinction (Supplementary Figure S2).

However, the OXT system might be only partially responsible for the observed behavioral differences between the strains. Neurotransmitters such as GABA, dopamine and serotonin, as well as other neuropeptides, such as vasopressin, CRF and NPS, are important modulators of anxiety-, stress- and fear-related behavior (Bludau et al., 2023; Grund and Neumann, 2019; Gryksa et al., 2023; Hauger et al., 2009; Jüngling et al., 2008; Lydiard, 2003; Pourhamzeh et al., 2022; Puglisi-Allegra and Andolina, 2015; Zarrindast and Khakpai, 2015; Zoicas et al., 2016). Thus, future studies need to reveal detailed differences in the activity of these neuroactive systems between BL6 and CD1 mice and their impact on the respective behavioral phenotype. For example, BL6 and CD1 mice might also differ in NPS signaling, since single nucleotide polymorphisms in the NPS receptor were found to be associated with increased anxiety traits in both humans and rodents (Donner et al., 2010; Grund and Neumann, 2019; Okamura et al., 2007; Slattery et al., 2015), while icv NPS infusion reversed SFC-induced social fear and reduced social defeat-induced social avoidance in male CD1 mice (Zoicas et al., 2016). Thus, various neurobiological systems—probably by interacting with each other—might create such substantial differences in innate behavior between the strains.

In conclusion, our data show profound behavioral differences between male BL6 and CD1 mice, with BL6 mice being more anxious, less social and more trauma-vulnerable compared to CD1 mice. Some of these behavioral strain differences were already present during adolescence but seemed to further manifest throughout development. In more detail, CD1 mice appeared to be less anxious and less vulnerable toward trauma and stress during adolescence and displayed a trajectory of reduced anxiety and resilience to pain and fear conditioning in adulthood. In contrast, the high trauma and stress vulnerability seen in adolescent BL6 mice remained throughout development and seemed to further manifest during transition into adulthood. Additionally, foot shock sensitivity and pain perception increased during adulthood in BL6, but not CD1 mice. These findings are of particular interest, since various psychopathologies have an early onset during puberty and depend on early life experiences and individual vulnerability (Broekman, 2011; Kessler et al., 2007; Polanczyk et al., 2015; Wright et al., 2020).

The OXT system appears to partially contribute to these behavioral phenotypes, since we found strain- and age-specific differences in OXTR binding in various relevant brain regions, whereas the number of OXT-positive neurons in the SON and PVN remained similar across all groups. Moreover, icv OXT did not mitigate the observed differences in socio-emotional behavior.

## Data Availability

The raw data supporting the conclusions of this article will be made available by the authors, without undue reservation.

## Supplementary material

SUPPLEMENTARY FIGURE S1 Strain difference in responses to repeated foot shock exposure of increasing intensities, thermal pain perception, and bodyweight in adolescent and adult male BL6 and CD1 mice. (A) Percent of adolescent and adult BL6 and CD1 mice responding to increasing foot shock intensities (0.05 mA, 0.1 mA, 0.15 mA, 0.2 mA, 0.3 mA, 0.5 mA, 0.7 mA, 1.0 mA) during the second day of the foot shock sensitivity comparison (FSSC). (B) Response score on the second day of FSSC of adolescent and adult BL6 and CD1 mice. (C) Percent of adolescent and adult BL6 and CD1 mice vocalizing during the first day of FSSC. (D) Paw withdrawal latency (sec) in the Hargreave’s plantar test (HPT) in adolescent and adult BL6 and CD1 mice. (E) Correlation between bodyweight (g) and paw withdrawal latency during HPT in adolescent (Adl) and adult (Adu) BL6 and CD1 mice. (F) Bodyweight of adolescent and adult BL6 and CD1 mice on the first day of FSSC and HPT. FSSC: *n* = 5/group. +*p* < 0.05, ++*p* < 0.01 adolescent BL6 vs CD1; **p* < 0.05, ***p* < 0.01 adult BL6 vs CD1; #*p* < 0.05, ##*p* < 0.01 adult vs adolescent BL6. HPT: *n* = 4–5/group, bodyweight *n* = 5/group. **p* < 0.05, ***p* < 0.01 BL6 vs CD1; #*p* < 0.05, ##*p* < 0.01 adult vs adolescent. Data represent mean ± SEM. SEM is not visible in graph whenever the value was too small. Each dot represents an individual mouse. For detailed statistics (see Supplementary Table S7).

SUPPLEMENTARY FIGURE S2 Effect of intracerebroventricular (icv) oxytocin (OXT) on social fear conditioning (SFC) in adult male BL6 mice. (A) Schematic representation of the experimental time plan for assessing the effect of icv OXT infusion on extinction of social fear. (B) CS-US pairings during acquisition of social fear, as well as time spent investigating non-social (ns) and social (s) stimuli during (C) extinction and (D) recall of social fear of mice infused with either vehicle (Veh) or a low dose of OXT (OXT_LD_=0.1 μg/2 μL). *n* = 8-9/group. Data represent mean ± SEM. For detailed statistics (see Supplementary Table S8).
