# Supplementary figures and images for: Beyond fur color: differences in socio-emotional behavior and the oxytocin system between male BL6 and CD1 mice in adolescence and adulthood
Source: Front Neurosci. 2024 Dec 9;18:1493619. doi: 10.3389/fnins.2024.1493619 (PMC11663876; doi:10.3389/fnins.2024.1493619)

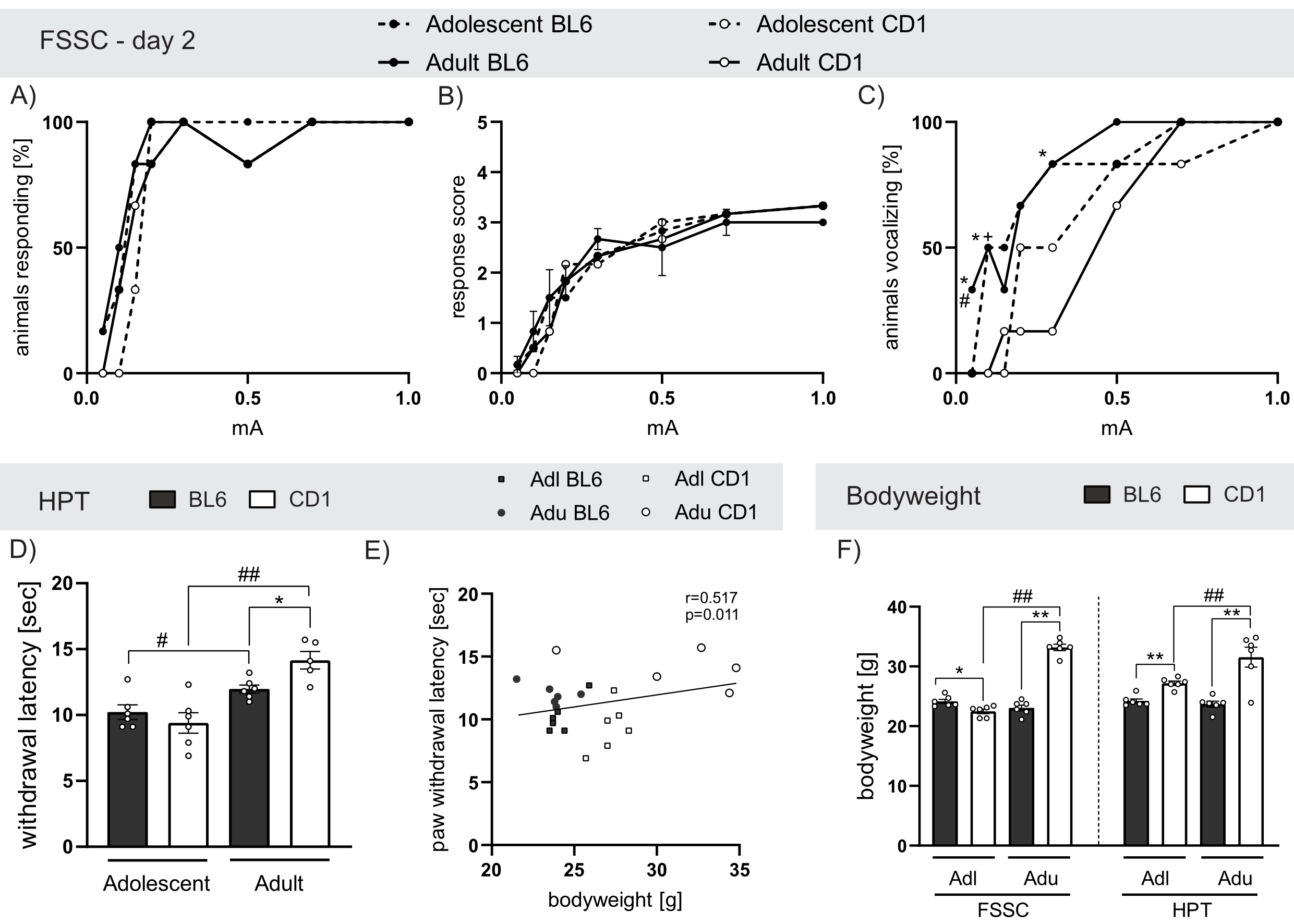

Supplement: SUPPLEMENTARY FIGURE S1 — Strain difference in responses to repeated foot shock exposure of increasing intensities, thermal pain perception, and bodyweight in adolescent and adult male BL6 and CD1 mice. (A) Percent of adolescent and adult BL6 and CD1 mice responding to increasing foot shock intensities (0.05 mA, 0.1 mA, 0.15 mA, 0.2 mA, 0.3 mA, 0.5 mA, 0.7 mA, 1.0 mA) during the second day of the foot shock sensitivity comparison (FSSC). (B) Response score on the second day of FSSC of adolescent and adult BL6 and CD1 mice. (C) Percent of adolescent and adult BL6 and CD1 mice vocalizing during the first day of FSSC. (D) Paw withdrawal latency (sec) in the Hargreave’s plantar test (HPT) in adolescent and adult BL6 and CD1 mice. (E) Correlation between bodyweight (g) and paw withdrawal latency during HPT in adolescent (Adl) and adult (Adu) BL6 and CD1 mice. (F) Bodyweight of adolescent and adult BL6 and CD1 mice on the first day of FSSC and HPT. FSSC: n = 5/group. +p < 0.05, ++p < 0.01 adolescent BL6 vs CD1; *p < 0.05, **p < 0.01 adult BL6 vs CD1; #p < 0.05, ##p < 0.01 adult vs adolescent BL6. HPT: n = 4–5/group, bodyweight n = 5/group. *p < 0.05, **p < 0.01 BL6 vs CD1; #p < 0.05, ##p < 0.01 adult vs adolescent. Data represent mean ± SEM. SEM is not visible in graph whenever the value was too small. Each dot represents an individual mouse. For detailed statistics (see Supplementary Table S7). [file Image_1.tiff]

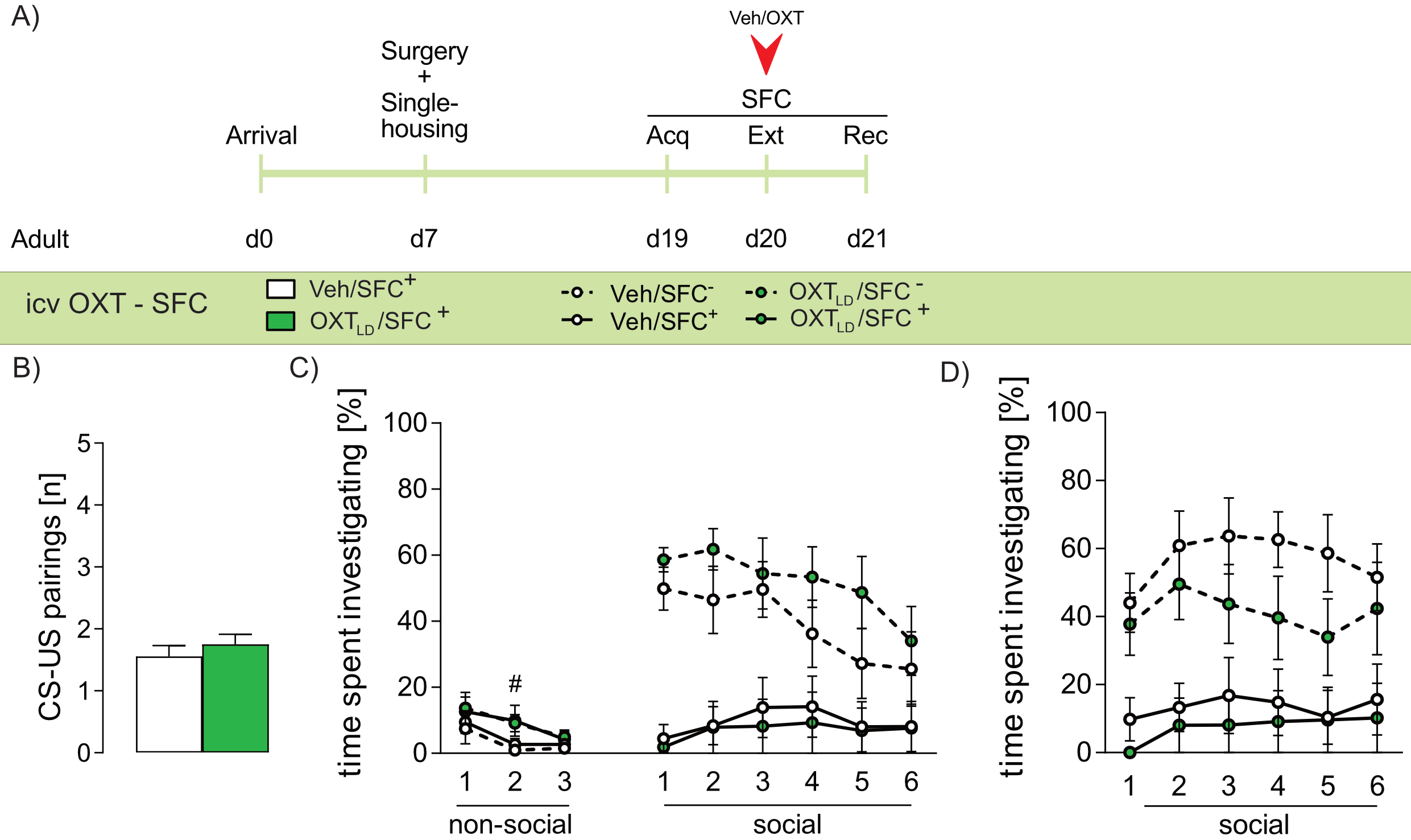

Supplement: SUPPLEMENTARY FIGURE S2 — Effect of intracerebroventricular (icv) oxytocin (OXT) on social fear conditioning (SFC) in adult male BL6 mice. (A) Schematic representation of the experimental time plan for assessing the effect of icv OXT infusion on extinction of social fear. (B) CS-US pairings during acquisition of social fear, as well as time spent investigating non-social (ns) and social (s) stimuli during (C) extinction and (D) recall of social fear of mice infused with either vehicle (Veh) or a low dose of OXT (OXTLD=0.1 μg/2 μL). n = 8-9/group. Data represent mean ± SEM. For detailed statistics (see Supplementary Table S8). [file Image_2.tiff]
