# Supplementary material for: Beyond fur color: differences in socio-emotional behavior and the oxytocin system between male BL6 and CD1 mice in adolescence and adulthood
Source: Front Neurosci. 2024 Dec 9;18:1493619. doi: 10.3389/fnins.2024.1493619 (PMC11663876; doi:10.3389/fnins.2024.1493619)
Supplement: Supplementary file 3 [file Table_1.pdf]

SUPPLEMENTARY TABLES

Table S1: Statistics of the behavioral differences of BL6 and CD1 mice on EPM, OFT and SPAT corresponding to Figure 1. Factor time represents non-social vs social stimulus presentations during SPAT; factor strain represents BL6 vs CD1 effects; factor age represents adolescent vs adult effects.

EPM	Two-way ANOVA	Figure 1B-D
Open arm entries [%]	(Age) $F_{1,36} = 1.494$	P = 0.230
	(Strain) $F_{1,36} = 42.663$	P < 0.001
	(Age x Strain) $F_{1,36} = 0.634$	P = 0.431
Time open arm [%]	(Age) $F_{1,36} = 9.968$	P = 0.003
	(Strain) $F_{1,36} = 19.092$	P < 0.001
	(Age x strain) $F_{1,36} = 7.081$	P = 0.012
Closed arm entries [n]	(Age) $F_{1,36} = 1.750$	P = 0.194
	(Strain) $F_{1,36} = 16.680$	P < 0.001
	(Age x strain) $F_{1,36} = 0.744$	P = 0.394
OFT	Two-way ANOVA	Figure 1E-F
Time center zone [s]	(Age) $F_{1,48} = 832.871$	P < 0.001
	(Strain) $F_{1,48} = 601.201$	P < 0.001
	(Age x strain) $F_{1,48} = 715.951$	P < 0.001
Distance travelled [m]	(Age) $F_{1,48} = 2.227$	P = 0.142
	(Strain) $F_{1,48} = 8.673$	P = 0.005
	(Age x strain) $F_{1,48} = 6.817$	P = 0.012
SPAT – Strain effects	Mixed Model ANOVA	Figure 1G
Investigation time [%]	(Time) $F_{1,18} = 44.306$	P < 0.001
Adolescent	(Strain) $F_{1,18} = 11.667$	P = 0.003
	(Time x strain) $F_{1,18} = 15.096$	P = 0.001
Investigation time [%]	(Time) $F_{1,33} = 31.561$	P < 0.001
Adult	(Strain) $F_{1,33} = 35.630$	P < 0.001
	(Time x strain) $F_{1,33} = 4.819$	P = 0.035
SPAT – Age effects	Mixed Model ANOVA	Figure 1G
Investigation time [%]	(Time) $F_{1,26} = 8.709$	P = 0.007
BL6	(Age) $F_{1,26} = 3.243$	P = 0.083
	(Time x age) $F_{1,26} = 0.052$	P = 0.822
Investigation time [%]	(Time) $F_{1,25} = 72.416$	P < 0.001
CD1	(Age) $F_{1,25} = 0.579$	P = 0.454
	(Time x age) $F_{1,25} = 2.249$	P = 0.146

Table S2: Statistics of the behavioral differences of BL6 and CD1 mice during CFC corresponding to Figure 2. Factor time represents CS-US pairings during acquisition and CS presentations during extinction; factor strain represents BL6 vs CD1 effects; factor age represents adolescent vs adult effects.

Adolescent CFC – Strain effects	Mixed Model ANOVA, Independent T-Test	Figure 3B-D
Acquisition	(Time) $F_{3,54}$ = 24.111	P < 0.001
	(Strain) $F_{1,18}$ = 29.033	P < 0.001
	(Time x strain) $F_{3,54}$ = 6.722	P = 0.001
Extinction	(Time) $F_{4.958,89.245}$ = 5.195	P < 0.001
	(Strain) $F_{1,18}$ = 110.649	P < 0.001
	(Time x strain) $F_{4.958,89.245}$ = 3.603	P = 0.005
Recall	T_{18} = 0.7973	P < 0.001
**Adult CFC – Strain effects**	**Mixed Model ANOVA, Independent T-Test**	**Figure 3E-G**
Acquisition	(Time) $F_{1.672,30.096}$ = 20.073	P < 0.001
	(Strain) $F_{1,18}$ = 37.197	P < 0.001
	(Time x strain) $F_{1.672,30.096}$ = 10.235	P < 0.001
Extinction	(Time) $F_{4.974,89.532}$ = 3.518	P = 0.006
	(Strain) $F_{1,18}$ = 116.962	P < 0.001
	(Time x strain) $F_{4.974,89.532}$ =0.616	P = 0.687
Recall	T_{18} = 11.732	P < 0.001
**BL6 CFC – Age effects**	**Mixed Model ANOVA, Independent T-Test**	**Figure 3B-G**
Acquisition	(Time) $F_{3,54}$ = 38.955	P < 0.001
	(Age) $F_{1,18}$ = 1.817	P = 0.194
	(Time x age) $F_{3,54}$ = 0.431	P = 0.732
Extinction	(Time) $F_{9,162}$ = 5.405	P < 0.001
	(Age) $F_{1,18}$ = 1.889	P = 0.186
	(Time x age) $F_{9,162}$ = 1.237	P = 0.276
Recall	T_{18} = -1.019	P = 0.322
**CD1 CFC – Age effects**	**Mixed Model ANOVA, Independent T-Test**	**Figure 3B-G**
Acquisition	(Time) $F_{1.981,35.652}$ = 8.095	P = 0.001
	(Age) $F_{1,18}$ = 0.083	P = 0.777
	(Time x age) $F_{1.981,35.652}$ = 1.873	P = 0.169
Extinction	(Time) $F_{4.530,81.544}$ = 5.539	P < 0.001
	(Age) $F_{1,18}$ = 0.477	P = 0.499
	(Time x age) $F_{4.530,81.544}$ =0.478	P = 0.775
Recall	T_{18} = 1.410	P = 0.176

Table S3: Statistics of the behavioral differences of BL6 and CD1 mice during SFC corresponding to Figure 3. Factor time represents repeated stimuli presentations during extinction and recall; factor strain represents BL6 vs CD1 effects; factor age represents adolescent vs adult effects.

Adolescent SFC – Strain effects	Mann-Whitney-U Test, Mixed Model ANOVA	Figure 4B-D
Acquisition	U = 52.5, z = -1.82	P = 0.101
Extinction non-social	(Time) $F_{1.838,80.868}$ = 47.271	P < 0.001
	(Strain) $F_{1,44}$ = 25.353	P < 0.001
	(Conditioning) $F_{1,44}$ = 0.535	P = 0.468
	(Time x strain) $F_{1.838,80.868}$ = 7.816	P = 0.001
	(Time x conditioning) $F_{1.838,80.868}$ = 0.344	P = 0.692
	(Strain x conditioning) $F_{1,44}$ = 1.817	P = 0.185
	(Time x strain x conditioning) $F_{1.838,80.868}$ = 0.628	P = 0.523
Extinction social	(Time) $F_{3.415,146.853}$ = 6.307	P < 0.001
	(Strain) $F_{1,43}$ = 23.264	P < 0.001
	(Conditioning) $F_{1,43}$ = 33.193	P < 0.001
	(Time x strain) $F_{3.415,146.853}$ = 0.843	P = 0.485
	(Time x conditioning) $F_{3.415,146.853}$ = 1.985	P = 0.110
	(Strain x conditioning) $F_{1,43}$ = 0.119	P = 0.731
	(Time x strain x conditioning) $F_{3.415,146.853}$ = 1.091	P = 0.359
Recall	(Time) $F_{3.039,133.728}$ = 6.592	P < 0.001
	(Strain) $F_{1,44}$ = 5.141	P = 0.028
	(Conditioning) $F_{1,44}$ = 6.949	P = 0.012
	(Time x strain) $F_{3.039,133.728}$ = 1.844	P = 0.142
	(Time x conditioning) $F_{3.039,133.728}$ = 1.315	P = 0.272
	(Strain x conditioning) $F_{1,44}$ = 0.364	P = 0.550
	(Time x strain x conditioning) $F_{3.039,133.728}$ = 1.222	P = 0.304
**Adult SFC – Strain effects**	**Mann-Whitney-U Test, Mixed Model ANOVA**	**Figure 4E-G**
Acquisition	U = 86, z = -2.43	P = 0.027
Extinction non-social	(Time) $F_{1.801,97.257}$ = 38.036	P < 0.001
	(Strain) $F_{1,54}$ = 1.501	P = 0.226
	(Conditioning) $F_{1,54}$ = 1.295	P = 0.260
	(Time x strain) $F_{1.801,97.257}$ = 0.192	P = 0.803
	(Time x conditioning) $F_{1.801,97.257}$ = 0.985	P = 0.370
	(Strain x conditioning) $F_{1,54}$ = 0.811	P = 0.372
	(Time x strain x conditioning) $F_{1.801,97.257}$ = 0.010	P = 0.985
Extinction social	(Time) $F_{2.559,138.175}$ = 11.195	P < 0.001
	(Strain) $F_{1,54}$ = 42.055	P < 0.001
	(Conditioning) $F_{1,54}$ = 36.297	P < 0.001
	(Time x strain) $F_{2.559,138.175}$ = 9.928	P < 0.001
	(Time x conditioning) $F_{2.559,138.175}$ = 8.691	P < 0.001
	(Strain x conditioning) $F_{1,54}$ = 9.343	P = 0.003
	(Time x strain x conditioning) $F_{2.559,138.175}$ = 1.725	P = 0.173
Recall	(Time) $F_{2.561,135.742}$ = 5.178	P = 0.003
	(Strain) $F_{1,53}$ = 40.507	P < 0.001
	(Conditioning) $F_{1,53}$ = 7.240	P = 0.010
	(Time x strain) $F_{2.561,135.742}$ = 2.997	P = 0.041
	(Time x conditioning) $F_{2.561,135.742}$ = 3.323	P = 0.028
	(Strain x conditioning) $F_{1,53}$ = 32.397	P < 0.001
	(Time x strain x conditioning) $F_{2.561,135.742}$ = 1.365	P = 0.258
**SFC – Strain and age effects**	**Two-way ANOVA**	**Figure 4B, E**
Acquisition	(Age) $F_{1,57}$ = 6.639	P = 0.013
	(Strain) $F_{1,57}$ = 6.928	P = 0.011
	(Age x strain) $F_{1,57}$ = 0.403	P = 0.528
**BL6 SFC – Age effects**	**Two-way ANOVA, Mixed Model ANOVA**	**Figure 4C-D, F-G**
Extinction non-social	(Time) $F_{1.676,85.487}$ = 30.230	P < 0.001
	(Age) $F_{1,51}$ = 4.596	P = 0.037
	(Conditioning) $F_{1,51}$ = 1.937	P = 0.170
	(Time x age) $F_{1.676,85.487}$ = 0.628	P = 0.509

	(Time x conditioning) $F_{1.676,85.487}$ = 0.499	P = 0.576
	(Age x conditioning) $F_{1,51}$ = 0.853	P = 0.360
	(Time x age x conditioning) $F_{1.676,85.487}$ = 0.048	P = 0.929
Extinction social	(Time) $F_{2.910,148.434}$ = 2.126	P = 0.101
	(Age) $F_{1,51}$ = 537	P = 0.467
	(Conditioning) $F_{1,51}$ = 61.535	P < 0.001
	(Time x age) $F_{2.910,148.434}$ = 1.662	P = 0.179
	(Time x conditioning) $F_{2.910,148.434}$ = 3.356	P = 0.022
	(Age x conditioning) $F_{1,51}$ = 0.358	P = 0.552
	(Time x age x conditioning) $F_{2.910,148.434}$ = 0.867	P = 0.457
Recall	(Time) $F_{2.608,132.988}$ = 5.134	P = 0.003
	(Age) $F_{1,51}$ = 2.847	P = 0.098
	(Conditioning) $F_{1,51}$ = 23.144	P < 0.001
	(Time x age) $F_{2.608,132.988}$ = 3.076	P = 0.036
	(Time x conditioning) $F_{2.608,132.988}$ = 3.270	P = 0.029
	(Age x conditioning) $F_{1,51}$ = 1.582	P = 0.214
	(Time x age x conditioning) $F_{2.608,132.988}$ = 0.723	P = 0.522
CD1 SFC – Age effects	Mann-Whitney-U Test, Mixed Model ANOVA	Figure 4C-D, F-G
Extinction non-social	(Time) $F_{1.884,88.536}$ = 55.155	P < 0.001
	(Age) $F_{1,47}$ = 0.083	P = 0.775
	(Conditioning) $F_{1,47}$ = 0.420	P = 0.520
	(Time x age) $F_{1.884,88.536}$ = 3.873	P = 0.027
	(Time x conditioning) $F_{1.884,88.536}$ = 0.437	P = 0.636
	(Age x conditioning) $F_{1,47}$ = 0.837	P = 0.365
	(Time x age x conditioning) $F_{1.884,88.536}$ = 0.848	P = 0.426
Extinction social	(Time) $F_{3.041,139.899}$ = 18.632	P < 0.001
	(Age) $F_{1,46}$ = 0.890	P = 0.350
	(Conditioning) $F_{1,46}$ = 17.378	P < 0.001
	(Time x age) $F_{3.041,139.899}$ = 1.251	P = 0.294
	(Time x conditioning) $F_{3.041,139.899}$ = 6.648	P < 0.001
	(Age x conditioning) $F_{1,46}$ = 2.769	P = 0.103
	(Time x age x conditioning) $F_{3.041,139.899}$ = 0.876	P = 0.457
Recall	(Time) $F_{2.840,130.632}$ = 7.362	P < 0.001
	(Age) $F_{1,46}$ = 0.319	P = 0.575
	(conditioning) $F_{1,46}$ = 0.00.	P = 0.970
	(Time x age) $F_{2.840,130.632}$ = 0.921	P = 0.429
	(Time x conditioning) $F_{2.840,130.632}$ = 2.760	P = 0.048
	(Age x conditioning) $F_{1,46}$ = 8.224	P = 0.006
	(Time x age x conditioning) $F_{2.840,130.632}$ = 0.381	P = 0.756

Table S4: Statistics of responses to foot shocks of increasing intensities of adult and adolescent BL6 and CD1 mice using FSSC corresponding to Figure 4. Factor intensity represents foot shocks at increasing intensities during FSIT; factor strain represents BL6 vs CD1 effects; factor age represents adolescent vs adult effects.

FSSC	Mixed Model ANOVA	Figure 2B-D
Response to shock	(Intensity) $F_{2.543,50.864} = 91.580$	$P < 0.001$
	(Age) $F_{1,20} = 13.065$	$P = 0.002$
	(Strain) $F_{1,20} = 36.290$	$P < 0.001$
	(Intensity x age) $F_{2.543,50.864} = 2.884$	$P = 0.053$
	(Intensity x strain) $F_{2.543,50.864} = 5.899$	$P = 0.003$
	(Age x strain) $F_{1,20} = 7.903$	$P = 0.011$
	(Intensity x age x strain) $F_{2.543,50.864} = 3.812$	$P = 0.020$
Response score	(Intensity) $F_{4.342,86.845} = 128.615$	$P < 0.001$
	(Age) $F_{1,20} = 4.515$	$P = 0.046$
	(Strain) $F_{1,20} = 3.457$	$P = 0.078$
	(Intensity x age) $F_{4.342,86.845} = 0.782$	$P = 0.550$
	(Intensity x strain) $F_{4.342,86.845} = 3.884$	$P = 0.005$
	(Age x strain) $F_{1,20} = 0.018$	$P = 0.896$
Vocalization	(Intensity x age x strain) $F_{4.342,86.845} = 3.523$	$P = 0.009$
	(Intensity) $F_{4.293,85.861} = 52.948$	$P < 0.001$
	(Age) $F_{1,20} = 3.451$	$P = 0.078$
	(Strain) $F_{1,20} = 37.254$	$P < 0.001$
	(Intensity x age) $F_{4.293,85.861} = 0.553$	$P = 0.709$
	(Intensity x strain) $F_{4.293,85.861} = 7.958$	$P < 0.001$
	(Age x strain) $F_{1,20} = 3.451$	$P = 0.078$
	(Intensity x age x strain) $F_{4.293,85.861} = 1.841$	$P = 0.124$

Table S5: Statistics of the molecular differences of adult BL6 and CD1 mice on the OXT system using RAR and IHC corresponding to Figure 5. Factor age presentations adolescent vs adult effects; factor strain represents BL6 vs CD1 effects.

RAR	Two-way ANOVA	Figure 5A
VMH	(Age) $F_{1,24} = 28.316$	$P < 0.001$
	(Strain) $F_{1,24} = 1.946$	$P = 0.176$
	(Age x strain) $F_{1,24} = 2.907$	$P = 0.101$
PAG	(Age) $F_{1,23} = 11.369$	$P = 0.003$
	(Strain) $F_{1,23} = 10.689$	$P = 0.003$
	(Age x strain) $F_{1,23} = 0.009$	$P = 0.924$
dLS	(Age) $F_{1,24} = 0.055$	$P = 0.817$
	(Strain) $F_{1,24} = 16.307$	$P < 0.001$
	(Age x strain) $F_{1,24} = 8.295$	$P = 0.008$
vLS	(Age) $F_{1,24} = 2.213$	$P = 0.150$
	(Strain) $F_{1,24} = 3.545$	$P = 0.072$
	(Age x strain) $F_{1,24} = 3.144$	$P = 0.089$
CeA	(Age) $F_{1,23} = 5.155$	$P = 0.033$
	(Strain) $F_{1,23} = 2.503$	$P = 0.127$
	(Age x strain) $F_{1,23} = 0.114$	$P = 0.739$
BLA	(Age) $F_{1,24} = 29.961$	$P < 0.001$
	(Strain) $F_{1,24} = 0.035$	$P = 0.853$
	(Age x strain) $F_{1,24} = 1.516$	$P = 0.230$
**IHC**	**Two-way ANOVA**	**Figure 5C**
PVN	(Age) $F_{1,15} = 2.631$	$P = 0.126$
	(Strain) $F_{1,15} = 0.158$	$P = 0.697$
	(Age x strain) $F_{1,15} = 0.025$	$P = 0.877$
SON	(Age) $F_{1,16} = 0.182$	$P = 0.675$
	(Strain) $F_{1,16} = 0.975$	$P = 0.338$
	(Age x strain) $F_{1,16} = 0.033$	$P = 0.858$

Table S6: Statistics of the behavioral differences of adult BL6 and CD1 mice following icv OXT injection on the EPM and SPAT corresponding to Figure 6. Factor strain represents BL6 vs CD1 effects; factor treatment represents Veh vs OXT_{LD} vs OXT_{HD} effects.

EPM	One-way ANOVA	Figure 6B-D
Open arm entries [%]	(BL6) $F_{2,33}$ = 0.070	P = 0.933
	(CD1) $F_{2,33}$ = 0.767	P = 0.472
Time open arm [%]	(BL6) $F_{2,33}$ = 2.83	P = 0.073
	(CD1) $F_{2,33}$ = 1.528	P = 0.232
Closed arm entries [n]	(BL6) $F_{2,33}$ = 3.59	P = 0.039
	(CD1) $F_{2,33}$ = 2.50	P = 0.098
**SPAT**	**Two-way ANOVA**	**Figure 6E**
Investigation time [%]	(Strain) $F_{1,71}$ = 43.9	P < 0.001
Non-social	(Treatment) $F_{2,71}$ = 5.72	P = 0.005
	(Strain x treatment) $F_{2,71}$ = 0.886	P = 0.417
Investigation time [%]	(Strain) $F_{1,71}$ = 39.4	P < 0.001
Social	(Treatment) $F_{2,71}$ = 2.13	P = 0.126
	(Strain x treatment) $F_{2,71}$ = 0.451	P = 0.639

Table S7: Statistics of thermal pain perception using HPT and bodyweight between adult and adolescent BL6 and CD1 mice corresponding to Supplementary Figure S1. Factor strain represents BL6 vs CD1 effects; factor age represents adolescent vs adult effects.

FSSC day 2	Mixed Model ANOVA	Figure S1A-C
Response to shock	(Intensity) $F_{3.888,77.753}$ = 36.184	P < 0.001
	(Age) $F_{1,20}$ = 0.656	P = 0.428
	(Strain) $F_{1,20}$ = 4.098	P = 0.056
	(Intensity x age) $F_{3.888,77.753}$ = 0.848	P = 0.496
	(Intensity x strain) $F_{3.888,77.753}$ = 1.237	P = 0.302
	(Age x strain) $F_{1,20}$ = 0.164	P = 0.690
	(Intensity x age x strain) $F_{3.888,77.753}$ = 0.671	P = 0.610
Response score	(Intensity) $F_{4.364,87.281}$ = 66.559	P < 0.001
	(Age) $F_{1,20}$ =	P = 0.950
	(Strain) $F_{1,20}$ =	P = 0.573
	(Intensity x age) $F_{4.364,87.281}$ = 0.622	P = 0.662
	(Intensity x strain) $F_{4.364,87.281}$ = 0.265	P = 0.265
	(Age x strain) $F_{1,20}$ =	P = 0.950
Vocalization	(Intensity x age x strain) $F_{4.364,87.281}$ = 0.259	P = 0.916
	(Intensity) $F_{3.615,72.300}$ = 28.194	P < 0.001
	(Age) $F_{1,20}$ = 0.016	P = 0.899
	(Strain) $F_{1,20}$ = 10.246	P = 0.004
	(Intensity x age) $F_{3.615,72.300}$ = 0.741	P = 0.554
	(Intensity x strain) $F_{3.615,72.300}$ = 2.007	P = 0.109
	(Age x strain) $F_{1,20}$ = 0.410	P = 0.529
	(Intensity x age x strain) $F_{3.615,72.300}$ = 1.029	P = 0.394
**HPT**	**Two-way ANOVA, pearson correlation**	**Figure S1D-E**
Paw withdrawal latency [sec]	(Age) $F_{1,19}$ = 29.395	P < 0.001
	(Strain) $F_{1,19}$ = 1.315	P = 0.266
	(Age x Strain) $F_{1,19}$ = 6.284	P = 0.021
Correlation paw withdrawal - bodyweight	r = 0.517, n = 23	P = 0.011
**Bodyweight**	**Two-way ANOVA**	**Figure S1F**
FSSC [g]	(Age) $F_{1,20}$ = 116.727	P < 0.001
	(Strain) $F_{1,20}$ = 90.835	P < 0.001
	(Age x Strain) $F_{1,20}$ = 176.243	P < 0.001
HPT [g]	(Age) $F_{1,20}$ = 29.395	P < 0.001
	(Strain) $F_{1,20}$ = 146.398	P < 0.001
	(Age x Strain) $F_{1,20}$ = 41.056	P < 0.001
**FSSC - bodyweight**	**Pearson correlation**	**Not illustrated**

0.05mA	no responses	
0.1mA	r = -0.215, n = 24	P = 0.314
0.15mA	r = -0.269, n = 24	P = 0.204
0.2mA	r = -0.253, n = 24	P = 0.232
0.3mA	r = 0.005, n = 24	P = 0.982
0.5mA	r = 0.304, n = 24	P = 0.148
0.7mA	r = 0.267, n = 24	P = 0.207
1.0mA	r = 0.152, n = 24	P = 0.479

Table S8: Statistics of the behavioral differences of adult BL6 mice following icv OXT injection during SFC extinction training corresponding to Supplementary Figure S2. Factor time represents repeated stimuli presentations during extinction and recall; factor treatment represents Veh vs OXT_{LD} effects; factor SFC represents SFC^- vs SFC^+ effects.

SFC	Mann-Whitney-U Test, Mixed Model ANOVA	Figure S2
Acquisition	U = 29, z = -0.812	P = 0.541
Extinction non-social	(Time) $F_{1.490,43,200}$ = 8.512	P = 0.002
	(OXT) $F_{1,29}$ = 4.715	P = 0.038
	(SFC) $F_{1,29}$ = 0.082	P = 0.777
	(Time x OXT) $F_{1.490,43,200}$ = 1.003	P = 0.354
	(Time x SFC) $F_{1.490,43,200}$ = 0.049	P = 0.910
	(OXT x SFC) $F_{1,29}$ = 0.188	P = 0.668
	(Time x OXT x SFC) $F_{1.490,43,200}$ = 0.040	P = 0.922
Extinction social	(Time) $F_{2.168,62.880}$ = 1.909	P = 0.153
	(OXT) $F_{1,29}$ = 1.492	P = 0.232
	(SFC) $F_{1,29}$ = 18.249	P < 0.001
	(Time x OXT) $F_{2.168,62.880}$ = 0.733	P = 0.495
	(Time x SFC) $F_{2.168,62.880}$ = 0.701	P = 0.511
	(OXT x SFC) $F_{1,29}$ = 0.316	P = 0.578
	(Time x OXT x SFC) $F_{2.168,62.880}$ = 1.250	P = 0.295
Recall	(Time) $F_{2.168,62.880}$ = 1.909	P = 0.153
	(OXT) $F_{1,29}$ = 1.492	P = 0.232
	(SFC) $F_{1,29}$ = 18.249	P < 0.001
	(Time x OXT) $F_{2.168,62.880}$ = 0.733	P = 0.495
	(Time x SFC) $F_{2.168,62.880}$ = 0.701	P = 0.511
	(OXT x SFC) $F_{1,29}$ = 0.316	P = 0.578
	(Time x OXT x SFC) $F_{2.168,62.880}$ = 1.250	P = 0.295
